# Genetic improvement and genomic resources of important cyprinid species: status and future perspectives for sustainable production

**DOI:** 10.3389/fgene.2024.1398084

**Published:** 2024-09-19

**Authors:** Kiran D. Rasal, Pokanti Vinay Kumar, Shasti Risha, Prachi Asgolkar, M. Harshavarthini, Arpit Acharya, Siba Shinde, Siyag Dhere, Avinash Rasal, Arvind Sonwane, Manoj Brahmane, Jitendra K. Sundaray, Naresh Nagpure

**Affiliations:** ^1^ ICAR - Central Institute of Fisheries Education, Mumbai, Maharashtra, India; ^2^ ICAR - Central Institute of Freshwater Aquaculture, Bhubaneswar, Odisha, India

**Keywords:** selective breeding, genetics, genomics, cyprinids, genomic selection

## Abstract

Cyprinid species are the most cultured aquatic species around the world in terms of quantity and total value. They account for 25% of global aquaculture production and significantly contribute to fulfilling the demand for fish food. The aquaculture of these species is facing severe concerns in terms of seed quality, rising feed costs, disease outbreaks, introgression of exotic species, environmental impacts, and anthropogenic activities. Numerous researchers have explored biological issues and potential methods to enhance cyprinid aquaculture. Selective breeding is extensively employed in cyprinid species to enhance specific traits like growth and disease resistance. In this context, we have discussed the efforts made to improve important cyprinid aquaculture practices through genetic and genomic approaches. The recent advances in DNA sequencing technologies and genomic tools have revolutionized the understanding of biological research. The generation of a complete genome and other genomic resources in cyprinid species has significantly strengthened molecular-level investigations into disease resistance, growth, reproduction, and adaptation to changing environments. We conducted a comprehensive review of genomic research in important cyprinid species, encompassing genome, transcriptome, proteome, metagenome, epigenome, etc. This review reveals that considerable data has been generated for cyprinid species. However, the seamless integration of this valuable data into genetic selection programs has yet to be achieved. In the upcoming years, genomic techniques, gene transfer, genome editing tools are expected to bring a paradigm shift in sustainable cyprinid aquaculture production. The comprehensive information presented here will offer insights for the cyprinid aquaculture research community.

## 1 Introduction

Aquaculture is a rapidly growing fastest food producing sectors and is vital for ensuring sustainable livelihoods, nutrition, and global food security (FAO, 2022). Over the past two decades, this sector has experienced substantial growth in global food output. As the world’s population approaches 9.7 billion by 2050, there is a rising demand for protein-rich food, making aquaculture crucial in meeting this challenge (Cottrell et al., 2018; Searchinger et al., 2018; Wang et al., 2023). The *per capita* consumption of fish has doubled from 9.0 kg in 1961 to 20.2 kg in 2020, contributing to almost 20% of animal protein intake in human diets (FAO, 2022). Aquaculture made a historic contribution to worldwide aquatic animal production in 2020, hitting 49.2 percent (FAO, 2022). This achievement was driven by approximately 500 cultured species, with 22 species contributing 75% of the total production (FAO, 2022; Naylor et al., 2021).

**FIGURE 1:**
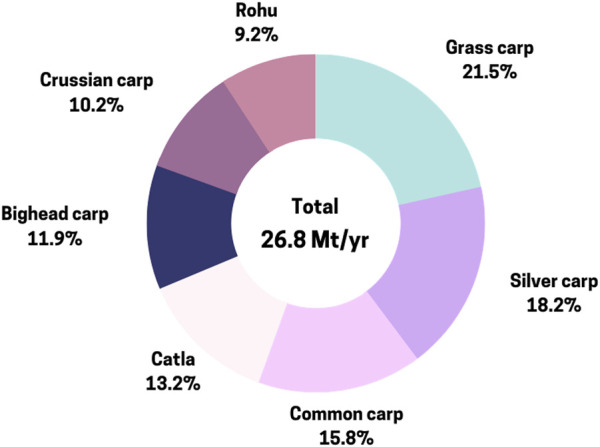
Annual production of cyprinid species with production above 1 Mt/yr (FAO/FishStatJ, 2022).

Global aquaculture production has surged from 17.3 million metric tons (MMT) in 1990 to 122.6 MMT in 2020, with significant contributions from seaweeds (4.2%), carps (27.8%), bivalves (2.8%), shrimps (16.4%), catfish (12.6%), and tilapia (15.9%) (FAO, 2022). Asia dominates this production, accounting for over 92%, led by China, which contributes 90% of aquaculture production in the region (Naylor et al., 2021). Global finfish production has risen from 20.8 MMT in 2000 to 57.5 MMT in 2020, with 13 finfish species representing around 72% of the total production. Carp production in Asia, particularly catla, rohu, grass carp, silver carp, common carp, bighead carp, and black carp, has experienced significant growth, reaching 27.6 MMT in 2020 (Figure 1) (FAO, 2022). Notably, catla (*Labeo catla)*, rohu (*Labeo rohita)*, grass carp (*Ctenopharyngodon idellus)*, silver carp (*Hypoththalmichthys molitrix)*, common carp (*Cyprinus carpio)*, bighead carp (*Hypophthalmichthys nobilis)*, and black carp (*Mylopharyngodon piceus*) account for roughly 50.6% of total inland aquaculture finfish output (FAO, 2022).

Cyprinid aquaculture in Asian countries exhibits a high level of diversity concerning the cultured species and production types and has undergone rapid evolution. The contribution of carp species in total production is dominated by grass carp, silver carp, common carp, catla, bighead carp*,* crucian carps (*Carassius s*pp.), and rohu etc. The feeding and breeding biology of these important carp species is well studied in Asian countries and is being used in aquaculture production through seed production and grow-out culture. In addition to these, various attempts were made to domesticate cyprinid species and genetically improve using selective breeding programs such as rohu in India and Bangladesh (Gjerde et al., 2002; Das Mahapatra et al., 2007) common carp in China, Indonesia, and Vietnam (Ninh et al., 2011) and silver barb (*Puntius gonionotus*) in Bangladesh and Thailand (Hussain and Mazid, 2002). In China, Ministry of Agriculture of China established a national committee for the examination and approval of original improved varieties of aquatic species and releases new aquatic species every year (Hu et al., 2021).

In order to meet global food demand, selective breeding of cyprinid species focuses on production factors like body growth and immunity. The systematic genetic improvement program in cyprinid species based on pedigreed populations and genetic merit resulted in an increased genetic gain of 12.5% per generation (Gjedrem and Robinson, 2014). The heritability for body weight in cyprinid species was observed to range from 0.2 to 0.4, which is considered good for the genetic selection program (Ninh et al., 2013). Initially, genetic variation in cyprinid species was studied based on morphometric characteristics, and later, new molecular genetic technologies were utilized for parentage assignment and stock variation analysis (Dey et al., 2010). The genetically improved cyprinid species demonstrated a significant improvement in the growth performance as compared to wild or local stock for the same species (Ponzoni et al., 2008; Das Mahapatra et al., 2007).

Molecular genetics and genomics studies in cyprinid species help to gain insight into the genetic basis of production and performance traits for implementing a genetic improvement program (Heras, 2021; Houston et al., 2020). Parentage analysis based on microsatellite markers supports the communal rearing of families of fish in the early stages, which reduces common environmental effects during the selection program (Ninh et al., 2013; Mahapatra et al., 2018). The genetic improvement program in cyprinid species such as rohu relied on phenotypic and pedigree data, which was restricted to a few production traits. During the last decade, advancements in molecular tools and genome sequencing enabled the development of diverse genomic resources for the important cyprinid species. These resources can be used for the enhancement of production efficiency, sustainability, product quality, and profitability of cyprinid culture by integrating them into the existing breeding programs.

In this paper, the progress made in genetic improvement programs and genomic resources developed at the genome, transcriptome, proteome, metagenome, epigenome, etc., in cyprinid species have been reviewed. The utilization of molecular markers such as microsatellite and single nucleotide polymorphisms (SNPs) developed in cyprinid species for production enhancement and evolutionary studies have been discussed. The complete genome and transcriptome analysis of important cyprinid species is a valuable resource for studying the genetic basis of characteristics and their inter-relationships. Functional annotations, genome-wide marker identification, linkage map construction, and genotyping might all be accomplished using a collection of genomic techniques and resources. Genome-editing tools could be used to elucidate the importance of epigenetic control of genes whose expression affects aquaculture performance. Cyprinid species’ genetic and genomic research is needed to shift towards more functional molecular marker discovery and understanding of gene regulatory networks for economically important traits. In addition, selection for feed conversion efficiency (FCE), reproductive parameters, and resilience to environmental stressors such as low DO, salinity, and temperature must be included in the cyprinid breeding programs. Further, the integration of omics data such as transcriptomics, proteomics, and metabolomics with a system biology approach would help to understand complex biological traits and their functional pathways, which contribute to a significant genetic variation among the stocks for enhancing cyprinid aquaculture production. This paper provides a comprehensive overview of cyprinid species genetic research and outlines the future research needs in their aquaculture, with a particular emphasis on genetics and genomics approaches.

## 2 Status of selective breeding program in cyprinids

Selective breeding in aquaculture is a long-term process that aims to genetically improve fish species for economically important traits such as growth rate, disease resistance, feed conversion efficiency, and reproductive fitness (Gjedrem and Robinson, 2014; Gjedrem and Baranski, 2010; Lind et al., 2012). This approach involves the meticulous selection of individuals based on their genetic parameters and pedigree information. Selective breeding aims to increase the prevalence of favorable alleles by allowing carriers to produce more offspring, leading to the gradual accumulation of genetic improvements over generations. This process relies on additive genetic variation, the hereditary contribution passed from one generation to the next. The history of selective breeding in aquaculture traces back to the 1920s, with early reports focusing on brook trout and common carp. Over the past 5 decades, selective breeding in aquatic species has shown remarkable success in achieving genetic improvement using family/individual selection, resulting in a significant 12.5% (average) increase in growth rate per generation. According to a report, genetically selected aquatic species contributed to 10%–20% of aquaculture production (FAO, 2022; Olesen et al., 2015).

In particular, cyprinids are an economically important species, and numerous selective breeding efforts has been carried out. The selective breeding programs in cyprinid species have primarily focused on enhancing the growth rate, followed by disease resistance and other traits. These programs have predominantly employed the family selection method for genetic improvement. The documented selective breeding programs for some of the commercially important cyprinid species worldwide are discussed below.

### 2.1 Common carp

The common carp, originating from Asia and Europe, is the third most globally introduced fish species and has been farmed since Roman times (Welcomme, 1988). Genetic improvement began in Hungary in 1962 under Dr. János Bakos, with the establishment of the common carp gene bank in Szarvas in 1963. Hybridization efforts, including mirror Szarvas 215, scaly Szarvas P. 31, and two-line scaly hybrid Szarvas P. 34, have demonstrated productivity surpassing parental lines by 20%–25% (Bakos and Gorda, 1995). The first instance of selective breeding among carp involved the common carp, aiming to enhance its growth rate by utilizing mass selection (Kirpichnikov and Faktorovich, 1974). Attempts to reduce the number of intermuscular bones of common carp by selection have not been successful in earlier times (Kossmann, 1972; Moav et al., 1975). In another study, Wohlfarth et al. (1975) observed that certain common carp populations from the river Amur showed resistance to dropsy, and crossing between local and Siberian wild carp led to the development of three stocks of Krasnodar common carp. These stocks were further utilized for commercial production through heterotic crossbreeding. The Jian carp were subjected to a six-generation mixed breeding procedure that included family selection, inter-line crossover, and gynogenesis. Individual selection for growth rate in common carp resulted in an increase in body weight over two generations in Vietnam, with estimated heritability ranging from 0.2 to 0.29 in just one of the three selected lines. Notably, after five generations of selection, the selected lines of common carp growth rate improved by 33% compared to the base population (Taniguchi et al., 1996).

It was observed significant growth differences among half-sibling families of common carp, with heritability estimates for growth at 0.47 (Brody et al., 1981). Early age body weight heritability was reported as zero (Vandeputte, 2003) and 0.12 (Nielsen et al., 2010). While a moderate heritability of 0.33 was noted for length, weight, and Fulton’s condition factor (K) at 2 months, and ranged between 0.39 and 0.49 at 4 months. A high heritability of 0.44 was reported for harvest body weight in Hungarian synthetic mirror carp over three generations (Vandeputte et al., 2008). Survival until harvest showed heritability of 0.2 (Nielsen et al., 2010). Significant genetic variation supports the notion that selective breeding can enhance carp growth and survival (Nielsen et al., 2010; Vandeputte et al., 2008; Vandeputte et al., 2004). Broodstock from the river Tisa and a Serbian fish farm displayed heritabilities of 0.34–0.45 in the first year and 0.44–0.49 in the second year for growth-related traits. Genetic correlations varied from 0.54–0.91 in the first year and 0.24–0.74 in the second year, generally remaining high. Close correlations between length and weight (0.80–0.98) suggest that indirect selection for length could facilitate genetic improvement for weight (Vandeputte et al., 2004; Vandeputte et al., 2008; Ninh et al., 2011). In contrast, it was reported relatively low genetic correlations (−0.54 to 0.47) for body weights recorded across different seasons in common carp, attributing the variance to temperature variations (Nielsen et al., 2010). Negative correlations were found between Fulton’s condition factor (K) and length (−0.38) and K and weight (−0.17) (Vandeputte et al., 2004).

Estimates for growth-related features in the Oujiang color common carp population have been reported (Wang et al., 2006) with heritabilities in the range of 0.14–0.30. The USSR developed the Ropsha carp, a fast-growing, cold-tolerant hybrid (Kirpichnikov and Faktorovich, 1974; Babouchkine and Tiews, 1987). Another study revealed a heritability of 0.44 for body weight in common carp based on data spanning three generations (Vandeputte et al., 2008). Researchers obtained a genetic gain of 7% relative to the base population of each generation after studying four generations of genetic selection for harvest body weight in common carp between 2004 and 2014. This study comprised the production, tagging, and rearing of 78 carp families over 20 months (Dong et al., 2015). A study on the heritability and genetic relationships between the weight, length, and height of common carp (*C. carpio* L.) throughout a three-year growing period was carried out (Spasić et al., 2010). The weight, length, and height of the 50 families of common carp that were born in 2007 were measured at tagging, first autumn, and second autumn. High heritability estimates based on univariate models were discovered for all traits during the second production year (Chen et al., 2022).

The high heritability for final weight (W3) and length (L3) in common carp (0.49, 0.50) and 0.34 ± 0.09 was reported for survival, suggesting successful selective breeding for these traits. Selective breeding programs for disease resistance for dropsy, in Krasnodar carp have been reported. It was estimated moderate heritability for body weight and low heritability for survival in common carp (Dong et al., 2015). A breeding program on Hungarian synthetic mirror carp resulted in heritabilities for body weight at 0.62 and total muscle fat at 0.23 to 0.41 (Prchal et al., 2018). High positive genetic correlations between growth and fillet yield indicated that selection for faster growth would improve fillet yield. In 2017, the base population of Amur mirror carp was established in the Czech Republic, and predictors for genetic improvement of slaughter yields were identified (Prchal et al., 2021). Earlier, low heritability for body weight for common carp at tagging was reported (Nielsen et al., 2010; Ninh et al., 2011). The predictors recorded on market-sized fish showed slightly better heritability (0.41, 0.44), genetic correlations to the slaughter yields (0.78–0.86), and expected genetic gains (1.29%–1.54%) of the slaughter yields than two-year old fish which suggests that the indirect selection for improved slaughter yields could be performed (Prchal et al., 2021). Overall, these studies indicated that significant genetic improvement in production traits such as body weight and processing traits were achieved in common carp through selective breeding.

Groundwater salinization is a global challenge, but it opens opportunities for cultivating saline-tolerant species like common carp, which can tolerate salinity up to 12 ppt. ICAR-CIFE in India has launched a selective breeding program to develop a faster-growing, low saline-tolerant common carp strain for inland saline aquaculture (Lalramnunsanga et al., 2024). The program uses diverse populations from across India, with genetic diversity assessed through truss morphometry and mitochondrial markers. The findings will aid in reducing inbreeding risks and support the genetic management of common carp in the ongoing breeding efforts.

### 2.2 Rohu

Rohu has become well-known as a promising aquaculture species in countries such as India, Bangladesh, Myanmar, and Pakistan. Due to effective induced breeding and domestication of this species, India and Bangladesh have used crossbreeding and selective breeding methods for genetic improvement. The ICAR-Central Institute of Freshwater Aquaculture (CIFA) in Bhubaneswar, Odisha, India, and the AKVAFORSK (Institute of Aquaculture Research) Norway jointly launched the selective breeding program for rohu in India in 1992 (Rasal and Sundaray, 2020). The process involved establishing a base population using six stocks collected from various rivers, including Ganga, Yamuna, Sutlej, Gomati, and Brahmaputra, along with a farmed stock of CIFA (Gjedrem and Robinson, 2014; Das Mahapatra et al., 2007). Full-sib families were generated using diallele crossing, and the fingerlings were tagged with a passive integrated transponder (PIT) for the evaluation of genetic parameters. Genetic improvement was carried out through a family selection method. The two complete diallele crosses involved five stocks of rohu carp to assess the impact of heterosis on growth and survival (Gjerde et al., 2002). The study reported that for harvest weight and survival, the total heterosis for each of the stock crosses was low or negative.

Over eight generations, an average genetic gain of 18% per generation was achieved in rohu, resulting in the development of a variety called “Jayanti” rohu (Mahapatra et al., 2016; Rasal et al., 2017a). The selective breeding program has continued, with 12 generations completed to date, and each generation consists of 50–60 families in each year class (Mahapatra et al., 2017). Furthermore, a disease resistance trait against the bacterial pathogen *Aeromonas hydrophila* was incorporated into the rohu selective breeding program, where arrays of single nucleotide polymorphisms (SNPs) were identified using transcriptome data from a study (Mahapatra et al., 2008; Robinson et al., 2014). Estimated heritabilities for challenge test survival obtained from the threshold model in Jayanti rohu is 0.11 ± 0.04 (Mahapatra et al., 2008). Experimental infection with *Aeromonas hydrophila* (9.55 × 106 cfu g-1 fish) through the intraperitoneal route produced higher survival in the resistant line (73.33%) as compared to the susceptible line (16.67%) (Sahoo et al., 2011). The resistant line showed 58% greater survival in the challenge test than the susceptible line (Sahoo et al., 2004). ICAR-CIFA received a Trade Mark Registration for “AhR Jayanti” rohu (*Aeromonas hydrophila*-resistant Jayanti rohu) in April 2022.

In Bangladesh, the WorldFish Carp Genetic Improvement Program (WFCGIP) initiated a selective breeding program for rohu focusing on the trait of harvest body weight in 2014 (Keus et al., 2017; Hamilton et al., 2019b). A base population developed utilizing 14 high-ranking full-sib families through family-based (pedigree-based) selection after procuring stocks from the Halda, Jamuna, and Padma rivers. After three generations (G3), significant realized genetic gains of 38.6% and 34.9% were observed for body harvest weight in the Jashore and Natore–Rajshahi populations, respectively (Hamilton et al., 2019b).

The genetic parameters for growth and survival in a rohu breeding program were studied under both mono and polyculture conditions. The estimated heritabilities (and common environmental effect for full-sibs, c^2^) across both production systems was 0.34 ± 0.10 (0.23 ± 0.04) for body weight at harvest. Genetic correlations between body weight at tagging and at sampling/harvest were of medium magnitude (0.38–0.49), while the correlation between body weight at sampling and harvest was very high (0.98), indicating that selection for increased harvest body weight would result in correlated genetic responses for early growth. The high magnitude of c^2^ (ranging from 0.66 to 0.78) for body weight at tagging emphasizes the need to standardize the rearing environment (Gjerde et al., 2019). A recent report suggested early selection of G1 individuals by visual identification and non-random sampling to enhance genetic gain in rohu harvest weight (Hamilton et al., 2022).

### 2.3 Catla

Catla, a highly significant aquaculture species, is predominantly cultivated in South Asia, often in combination with other species (Penman et al., 2005). However, factors including excessive levels of inbreeding, unchecked interspecific hybridization, and negative selection have traditionally had an impact on the quality of the seed produced in hatcheries (Khan et al., 2018). A project financed by the United States Agency for International Development (USAID) was started in 2012 to address these issues. Fertilized spawn from the Halda, Jamuna, and Padma (Ganges) rivers was gathered as part of this initiative to supply Bangladeshi catla hatcheries with genetically varied and non-inbred broodstock (Keus et al., 2017). Fin-clipping the fish and genotyping them with the DArTseq technology led to the discovery of 3048 single nucleotide polymorphisms (SNPs) and 4,726 silicoDArT markers. Intriguingly, the percentage of those without common putative parents was lower in the Jamuna and Padma fish sources, at 18.4% and 8.0%, respectively, compared to the Halda source, with 46.8% (Hamilton et al., 2019a).

The ICAR-Central Institute of Freshwater Aquaculture (ICAR-CIFA) initiated a selective breeding program on Catla (*Labeo catla*) in 2010 to enhance body weight at harvest, addressing the needs of the fish farming community. For the base population, nine strains/populations of *L. catla,* including two riverine strains (Ganga and Subernarekha) from different geographical regions (West Bengal, Bihar, Odisha, Andhra Pradesh, and Uttar Pradesh), were collected (Mahapatra et al., 2018). Phenotypic information on growth traits and microsatellite markers was utilized to infer relationships within and between strains. Combined family selection method was employed to select superior animals for each generation based on their breeding value (Mahapatra et al., 2016). A nested mating design, using the dry stripping method, produced full-sib and half-sib families. PIT tagging was conducted on enhanced catla fingerlings (10–150 g), revealing that the most effective size for PIT tagging in catla was 20–30 g (Mahapatra et al., 2022). A mixed linear animal model, with a pond as a fixed effect, was employed to estimate genetic parameters, and heritability for body weight at harvest was estimated to be 0.29 ± 0.03. After two generations of selection, a 15% genetic gain per generation was achieved in genetically improved catla (Mahapatra et al., 2022). A study examining the genotype-by-culture system interaction in catla revealed no evidence of heterosis or differences between their genetic groups concerning harvest weight or survival (Hamilton et al., 2023a).

### 2.4 Silver carp

Silver carp is the second-most significant species of farmed fish (FAO, 2020). The yearly silver carp production in Bangladesh is around 0.2 MT (DoF, 2019). WorldFish conducted a family-based (i.e., pedigree-based) genetic improvement initiative to enhance the growth rate in polyculture production systems, thereby improving the genetic quality of silver carp (Hamilton et al., 2021). Also, there exists a substantial additive genetic variation in the growth rate of about 43% greater average weight and 11% higher average length observed in Bangladeshi silver carp (Gheyas et al., 2009).

As “candidate founders” for a family-based genetic enhancement initiative in 2015–16, WorldFish obtained silver carp individuals from 21 Bangladeshi hatcheries. For generating the base population, 230 broodfish were used. The mean pedigree-derived additive genetic relationship between actual founders was low (0.0093) based on microsatellite markers, indicating that relationships between actual founders are unlikely to have a significant influence on future parent selection, mating choices, or inbreeding rates (Hamilton et al., 2021). The genetic variation and correlations between harvestage and a set of secondary traits (i.e., gill raker sponginess, gut length, the extent of overlap of pectoral and pelvic fins, presence of Lernaea and prevalence of red spots - sites of inflammation/hemorrhaging) were quantified in silver carp. The result suggested to have no significant correlation between the selected traits (Hamilton et al., 2023b). In silver carp, a higher heritability estimate of 0.67 at 6 months of age was reported by (Gheyas et al., 2009) Gheyas and his colleagues. For other traits, Hamilton and his team found moderate heritabilities of 0.24 for harvest-age weight and 0.22 for pectoral/pelvic fin overlap. In contrast, lower heritabilities were observed for the gill raker score (0.12) and relative gut length (0.09). Notably, there were no genetic correlations between harvest-age weight and secondary traits such as gill raker sponginess, gut length as a ratio of standard length, extent of overlap of pectoral and pelvic fins, presence of Lernaea, and prevalence of red spots (Hamilton et al., 2023b). This suggests that indirect selection or correlated responses cannot be obtained for these two traits.

### 2.5 Bighead carp

The bighead carpis an important freshwater food cyprinid species predominantly in China, contributing to over 90% of the world’s annual production, which exceeded 3.2 million tons in 2020 (FAO, 2020). It is renowned for its large size, high fecundity, and long maturity age (4–5 Years). Notably, its head makes up approximately 34% of the whole fish, contributing to its higher market value due to its rich nutritional and culinary qualities (Hong et al., 2013). It is a major aquaculture species in China and is selectively bred for faster growth and larger head size (Zhou et al., 2021b). To enhance its economic value, selective breeding for fast growth and larger head size is considered an important goal in bighead carp aquaculture (Zhou et al., 2021b). Genetic analysis of bighead carp populations (eight) in the Yangtze River using 15 microsatellite DNA markers revealed high diversity, weak differentiation among populations (Fst = 0.02, p < 0.01), and individual-level genetic admixture (Zhu et al., 2022). A genetic linkage map for bighead carp was constructed with 905 microsatellites assigned to 24 linkage groups. QTL mapping identified significant and suggestive QTL associated with growth traits on LG9 and LG17, explaining 18.6%–25.5% of phenotypic variance (Liu et al., 2016). A cross-breeding program conducted in Hungary among cyprinids revealed that common carp and bighead carp had good growth rates (BaKos et al., 1978). The reciprocal crosses between silver carp and bighead carp have higher survival, yield, and tameness than both the parents (Issa et al., 1986), and these hybrids are fertile but have low efficiency in controlling microalgal blooms due to the larger spacing of the gill filter apparatus (Marian et al., 1986).

A parentage test was conducted in bighead carp using ten microsatellite markers, resulting in a high success rate of 98.96% and 100% in two designed mating groups. These markers exhibited high polymorphism and heterozygosity (Zhang et al., 2019a). In a study on bighead carp, a high-density genetic map was created with 3,121 SNP markers from 117 individuals in an F1 family. This map spanned 2,341.2 cM with an average marker interval of 0.7 cM. Heritability estimates for body weight (BW) and standard length (SL) in 30-day-old bighead carp were 0.47 and 0.49, respectively. The study detected 37 suggestive QTL for five growth-related traits, explaining phenotypic variance from 15% to 38% (Fu et al., 2016a). The long sexual maturity time of bighead carp (4–5 years or more) poses challenges for selective breeding efforts, prompting the need for efficient breeding strategies. In a selective breeding program involving bighead carp (840 fishes), researchers estimated the heritability of growth-related traits and identified growth-related QTL using resequencing data (Chen et al., 2023). High phenotypic (0.70–0.95) and genetic (0.77–0.97) correlations were found among all growth-related traits. The estimated heritability values were as follows: 0.2 ± 0.11 for body-weight, 0.28 ± 0.14 for body-height, 0.32 ± 0.16 for head-length, 0.14 ± 0.11 for total length, and 0.12 ± 0.09 for body-length in seven-month-old bighead carp. These findings provide valuable insights for improving the efficiency of bighead carp breeding and production.

### 2.6 Grass carp

Grass carp is a highly productive freshwater fish in China, with local adaptation to diverse environments (Shen et al., 2019). It is primarily cultivated through pond culture, accounting for 71% of freshwater aquaculture production (Wang et al., 2015). Studies have focused on genetic parameters for growth, disease-resistance, and muscle protein and fat content (Zhang et al., 2019c; Fu et al., 2015; Xie et al., 2018). In previous studies, it was found that heritability estimates for growth traits ranged from 0.24 to 0.38, indicating a significant genetic variation (*P* < 0.01). High genetic and phenotypic correlations were observed among growth traits (0.81–0.99, *P* < 0.01), suggesting the potential for significant genetic improvement in these traits through selective breeding (Fu et al., 2016b). Similarly, in another study, heritability estimates were determined for early growth traits, and it was observed 0.304 for standard length (SL), 0.307 for body-weight (BW), and 0.150 for condition factor (K) at 40 days post-hatch (dph) (*P* < 0.05). Significant genetic and phenotypic correlations were observed between SL and BW, with values of 0.83 and 0.95, respectively (Fu et al., 2015).

For the selective breeding of grass carp, researchers identified 15 growth-related quantitative trait loci (QTLs) across 7 linkage-groups. These QTLs were associated with body-weight, body- length, body-height, and body-width based on microsatellite data. They explained varying percentages of phenotypic variance and provided valuable genetic insights for selective breeding (Yu et al., 2020). In the recent study on reducing intramuscular bones (IBs) in *C. idella* through selective breeding, researchers used high polymorphic microsatellite loci. The heritability of IBs number was also low at 0.137, suggesting that low heritability signifies that the genetic influence on IBs number was modest, making it challenging to substantially alter this trait through selective breeding efforts (Xiong et al., 2023). The genetic correlation among growth traits in grass carp ranged from 0.81 to 0.99 (Fu et al., 2016b; Rothbard and Wohlfarth, 1993). Rothbard and Wohlfarth (1993) reported albinotic and red-colored strains for grass carp (Rothbard and Wohlfarth, 1993). Wang et al. (2022c) demonstrated that disease-resistant grass carp (DR-GC) exhibited a 22.18% higher survival rate and a 16.31% faster growth rate than normal grass carp (GC) (Wang et al., 2022a). They also reported that the input–output ratio of DR-GC to GC is 1.00:1.30, showcasing significant cost-saving, efficiency-increasing, and higher-breeding benefits in DR-GC (Wang et al., 2022b).

Hybrids of grass carp and bighead carp were reported to be triploid (Márián and Krasznai, 1979; Beck et al., 1980), with diploids showing reduced viability. Triploids are preferred when both controlling aquatic vegetation and preventing its naturalization in ecosystems are necessary (Shireman and Smith, 1983).

### 2.7 Crucian carp

Crucian carp underwent domestication in 12th century in China (Kirpichnikov, 1981). Goldfish, reared during the Tang and Song Dynasties, became symbolic (Chen, 1956; Chen et al., 2020). Initially named *Cyprinus auratus,* it was later renamed *Carassius auratus,* closer to Crucian carp (Komiyama et al., 2009). Phylogenetic analysis showed Japanese goldfish originated from the Chinese Crucian carp “Gibelio” (Komiyama et al., 2009). Comet, a Goldfish variant, was produced in 1872 in the United States (Smartt, 2001). Various color and morphology variants developed through crossbreeding and selection (Tave, 1986; Kirpichnikov, 1981; Yamamoto, 1973). Selection of goldfish focused on features like double or triple tails (Komiyama et al., 2009). Pengze crucian carp, bred since the 1980s, underwent over 10 generations of selection (Zhou and Gui, 2002; Zhou et al., 2000). Artificial selection for goldfish traits, such as dorsal fin loss and diverse eye features, occurred independently (Komiyama et al., 2009). Currently, over 180 variants and 70 strains are produced globally (Nasu and Ohuchi, 2016). Some goldfish strains exhibit unique phenotypes (Omori and Kon, 2019). Intergeneric hybrids between common carp and crucian carp show male sterility but accelerated growth. Hybrids between crucian carp and grass carp hybrids are triploid (Kasama and Kobayasi, 1989). Intergeneric hybrids between *Carassius auratus* and *Gnathopogon elongatus elongates* resemble *C. auratus* (Suzuki, 1963).

### 2.8 Wuchang bream

The blunt snout bream (*Megalobrama amblycephala*), also known as Wuchang bream, is an endemic species in China. It has been a major economic species in Chinese freshwater aquaculture due to its high larval survival rate, rapid growth, and tender flesh (Wang and Gao, 2018). Selective breeding programs have focused on growth traits and disease resistance (Xun et al., 2023; Zhang et al., 2014; Xiong et al., 2019; Luo et al., 2014b). The study estimated heritabilities for growth-related traits (body-weight, total length, body-length, and body-height) in Wuchang Bream using a microsatellite-based pedigree approach. Heritabilities were found to be high (0.5–0.6), and there were strong genetic correlations among these traits. Selective breeding for growth in this species, particularly focusing on body-length, is considered feasible based on the findings (Luo et al., 2014a). Additionally, the selectively bred F5 population of Wuchang bream exhibited improved tolerance to hypoxic stress (Wu et al., 2020). In previous studies, the heritability of intramuscular bones (IB) in blunt snout bream was assessed for reducing IBs. This work resulted in a wide range of IB counts in the test population based on microsatellite data. The genetic correlation between IB numbers in different sections was high (0.9), while the phenotypic correlation was low (0.2). The heritability of IB numbers was medium (0.2–0.3) for some sections, indicating the potential for selective breeding to reduce IBs in this fish species (Xiong et al., 2019).

### 2.9 Black carp

The black carp is a large and fast-growing cyprinid, and native to eastern Asia (Feng et al., 2006). It is a preferred species for biological control of molluscans (Pieterse et al., 2017). In order to establish molecular selective breeding in black carp, QTL for four growth-related traits were identified, and a high correlation coefficient of 0.984, 0.97, 0.958, and 0.946 has been observed between the traits such as BL and BWI, BWE, and BL, BWE and BH, BWE and BWI, respectively (Guo et al., 2022). These studies reveal that selection for one of the four traits may also result in the improvement of others. A total of 10%–15% phenotypic variation was observed based on QTL. The QTL regions controlling the growth-related traits were concentrated on certain linkage groups such as LG10, LG17, and LG20, and suggested that LG17 could be exploited for MAS in the selective breeding programs of black carp (Guo et al., 2022).

### 2.10 Tench

Tench (*Tinca* L.) is one of the original European cyprinid species and is reared in farm ponds, either in monoculture or polyculture (Svobodova and Kolarova, 2004). The selective breeding and domestication of tench was started in the former Czech, and in addition to the five original strains, new strains from Hungary, France and Romania have also been introduced (Svobodova and Kolarova, 2004). The formation of 6 tench strains using a diallel crossing system among 6 strains from 4 populations has been carried out (Kvasnicka et al., 1998). Various color mutants has been reported in tench (Kvasnicka et al., 1998; Rothbard et al., 2010).

## 3 Genomics selection and genome-wide association studies (GWAS) in cyprinid species

Genomic selection (GS) is an advanced molecular breeding method in aquaculture that uses markers, like single-nucleotide polymorphisms (SNPs), to enhance the accuracy of breeding values. This method harnesses genomic relationships and markers associated with essential genes, with its application steadily expanding across various aquaculture species. GS demonstrates efficacy in refining critical traits such as growth and disease resistance, driven by the overarching objective of bolstering sustainability and fortifying resilience against the impacts of climate change. Despite its considerable potential, the widespread integration of GS remains somewhat constrained, though the prospect of cost-effective strategies holds promise for broader implementation.

Certain desirable traits (i.e., feed efficiency, disease resistance, fillet/carcass yields, and flesh quality) are challenging to measure directly and require evaluations of siblings. In contrast to classical pedigree-based selection, GS excels at capturing within-family genetic variation, thereby enhancing the overall genetic response. GS also endeavours to reduce the generation interval and mitigate the challenges associated with inbreeding. The comprehensive GS program encompasses the establishment of breeding populations, individual phenotyping, estimation of genomic estimated breeding values (gEBV), trait-based selection, and the development of training populations involving genotyping. The single-step Genomic Best Linear Unbiased Prediction (GBLUP) method optimally utilizes available information for precise predictions.

Thus, genomic selection involves selecting individuals based on estimated breeding values (gEBV) derived from genotypic (genome-wide genetic markers) and phenotypic information (Yáñez et al., 2015). The use of genomic selection in significant aquaculture species has been made feasible with high-quality reference genomes and genome-wide markers. The development of SNP arrays and genotyping methods has enabled the integration of the findings into genomic selection programs for aquaculture species. In particular, for qualitative characteristics like disease resistance and stress tolerance, these techniques have a substantial potential to increase genetic gain and improve the accuracy of anticipated breeding values, as noted in several review articles (Zenger et al., 2019; Houston et al., 2020; Yanez et al., 2023; Cao et al., 2020). Several studies hypothesized that genome-wide association studies (GWAS) may be used to assess the underlying genetic variation of complex traits.

Genomic selection is a recent advancement being utilized in aquaculture species, which have available marker panels/arrays (Yanez et al., 2023). Initially, genotype by sequencing with 12,311 SNP markers was applied in common carp for evaluating the potential of GS for growth improvement by estimating breeding values for 1,425 juvenile carp. The heritability for body length at 120 days was found to be 0.33 (Palaiokostas et al., 2018). The study led to an 18% improvement in the genomic prediction accuracy of estimated breeding values (EBV). To enhance the prediction accuracy of estimated breeding values and genetic gain against Koi Herpesvirus disease (KHVD) in common carp, the genomic selection was conducted on 1,425 juveniles using phenotypic and SNP markers. It was discovered that a QTL on LG 44 accounted for 7% of the genetic variance in KHVD resistance. Comparing the genetic prediction to the pedigree BLUP technique, the accuracy ranged from 8% to 18% higher (Palaiokostas et al., 2019). In yellow river carp, genomic selection using an SNPs array increased growth-related trait prediction by 2% (Wang et al., 2021a). In Amur mirror carp, low-density SNP panels (218 SNPs) showed high heritability (0.42–0.96) for KHVD resistance in selected populations (Prchal et al., 2023). This shows that genomic selection strategies for enhancing KHVD resistance in carps can be successful when using low-density SNP arrays.

The role of genomics in enhancing various traits in aquaculture has been explored through several studies. In crucian carp, a study utilizing 8 K SNP markers with 50 linkage groups (LGs) demonstrated their association with feed conversion efficiency, explaining 14.0%–20.9% of phenotypic variations (Pang et al., 2017). Gibel carp (*C. gibelio*) showcased genomic markers linked to feed conversion efficiency, indicating potential reductions in fishmeal consumption and improved fish growth (Shi et al., 2022). In common carp, the identification of 28 K SNP markers associated with Feed Conversion Efficiency (FCE) and genotyping with the carp 250 K SNP revealed nine key genes linked with feed efficiency traits (Zhang et al., 2021). Additionally, QTL mapping and GWAS in a Mirror carp family challenged with CyHV-3 highlighted the potential roles of mTOR, herpes simplex infection pathways, and homoeologous expression divergence in immune responses (Chen et al., 2022). Genome-wide studies on common carp families identified significant loci and candidate genes associated with muscle fat content (Zheng et al., 2016). In yellow river carp, GWAS associated with abnormal scale patterns, using the common carp 250 K SNP, identified key mutations in the fibroblast growth factor receptor 1 a1 (*fgfr1a1*) gene, suggesting potential for selection based on these mutations (Zhou and Gui, 2018). Additional, GWAS studies in common carp focused on muscle fat content and abdominal fat traits, revealing the complex genetic basis of fat metabolism and deposition (Zheng et al., 2016). In grass carp, GWAS with a 21 K array showed positive genetic correlation (0.9) and moderate to high heritability (0.4–0.5) among growth-related traits (Hao et al., 2023). Similarly, in bighead carp, GWAS associated with head-size and head-shape traits identified six significant SNP markers, suggesting their potential use in selection programs (Zhou et al., 2021b). Skin color, a crucial trait in cultured species, was investigated in yellow river carp through GWAS using the common carp 250 K SNP array, identifying eighteen significant SNP markers and shedding light on the genetic basis of abnormal skin coloration (Jiang et al., 2022a).

Despite challenges such as genotyping costs and limitations in the size of the reference population, genomic selection (GS) stands out as a promising approach for optimizing aquaculture breeding programs. The implementation process involves constructing a genomic prediction equation, estimating genomic breeding values (gEBV), and selecting candidates based on these values. Rigorous independent validation ensures the accuracy of predictions, facilitating the identification of the most suitable animals for breeding. However, GS faces constraints related to mating design, the number and size of families, the number of generation intervals, genome size, marker panel density, and selection methods. Parameters like genotype by environment interactions, the application of heterosis in multi-trait selection, realistic LD, modeling, and index selection have been largely overlooked in simulation studies to date. These aspects warrant investigation to efficiently optimize potential GS breeding programs.

## 4 Genomic resources in cyprinid species

### 4.1 Whole-genome and mitochondrial genome sequence of important cyprinids

The field of aquaculture and fisheries has experienced a revolution in genomic research owing to advances in sequencing technology. A genome encompasses all the DNA content, both coding and non-coding, within an organism. The year 2011 marked a significant milestone when the first genome of Atlantic cod was successfully sequenced (Star et al., 2011). Since then, the genomes of many significant aquaculture species have been sequenced and made available in public databases (Star et al., 2011; Lu and Luo, 2020). The progress in genome sequencing of important fish species has been extensively reviewed by various authors (Lu and Luo, 2020; Fan et al., 2020). In 2009, the Fish 10 K Genome Project was launched with the ambitious goal of sequencing and assembling genomes of approximately 10,000 vertebrate species, including 4,000 fish species (Bernardi et al., 2012).

**FIGURE 2:**
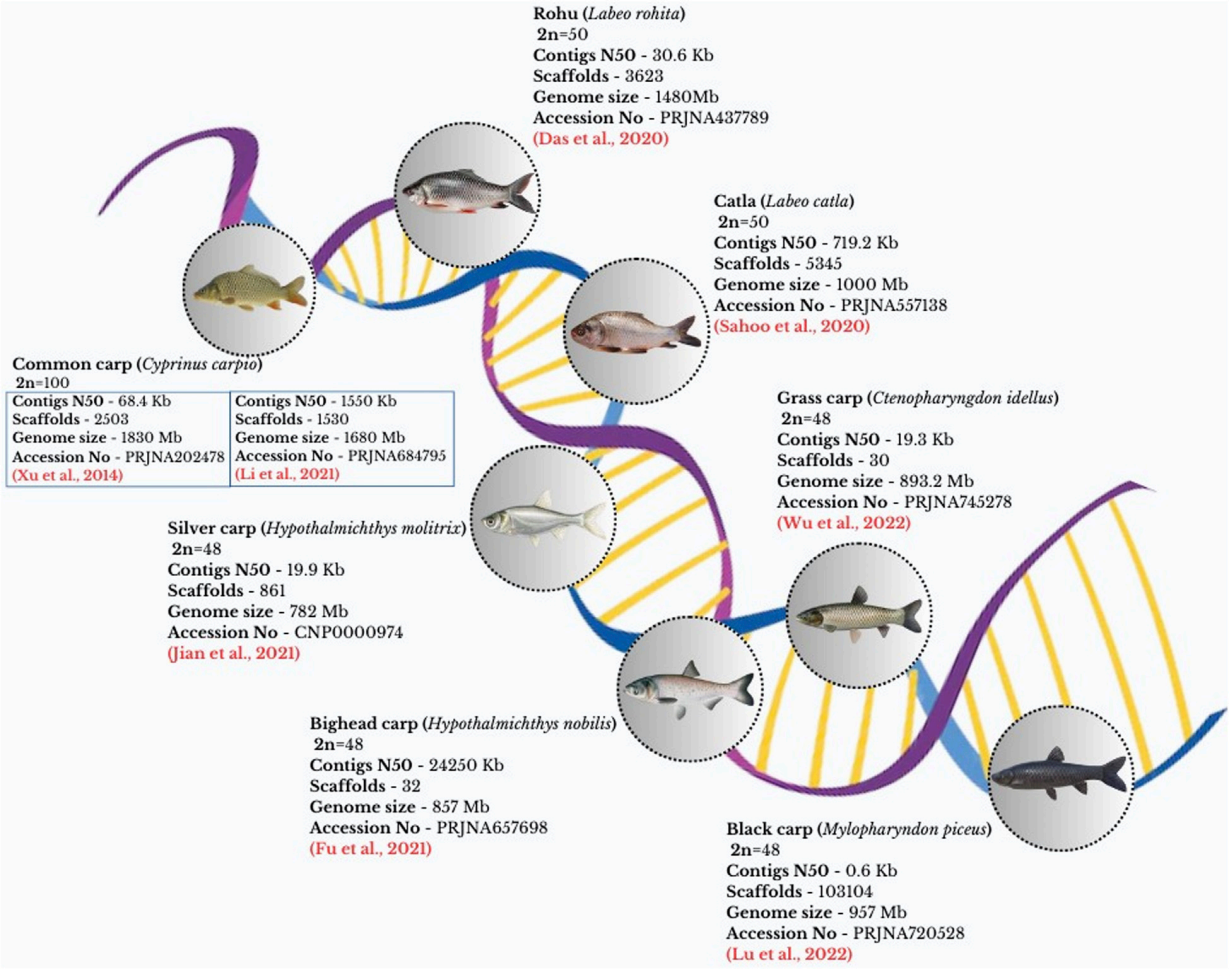
Whole genome sequence of important cyprinid species indicating genome size, Contigs N50, Scaffolds number.

Among the cyprinid species, seven important carp genomes have been sequenced, as depicted in Figure 2, and are available for various applications such as growth, development, disease resistance, reproduction, etc. The domesticated gynogenetic *C. carpio* (strain Songpu) genome was successfully sequenced in 2014, making it the first carp genome based on several NGS technologies and a hybrid assembly method. This genome was analyzed to have a GC content of 37.0%, which was somewhat greater than *Danio rerio*’s but much lower than the GC contents of other sequenced teleost genomes. In addition, scientists used resequencing on four wild and six domestic strains from Asia and Europe to explore the existing genetic diversity (Xu et al., 2014b).

The grass carp’s genome was sequenced and found to be 1.07 Gb for a female and 0.9 Gb for a male. The genome’s N50 was 6.5 Mb, distributed across 114 scaffolds (Wang et al., 2015). Nevertheless, to address the low continuity level and absence of chromosomal-level assembly, resequencing was performed using the PacBio system (Wu et al., 2022a). Sequencing of the double-haploid silver carp and bighead carp’s genomes produced 837 Mb and 845 Mb draft genome assemblies, respectively. According to the results of the phylogenetic study, the silver and bighead carp formed a clade, and their estimated period of divergence was around 3.6 million years ago (Jian et al., 2021).

A total of 1.01 Gb assembled genome size was reported for *Labeo catla*. This serves as an important resource for research on the comparative biology, evolution, and genomes of cyprinid species (Sahoo et al., 2020). In the case of Rohu, a draft genome of 1.48 Gb, along with five million SNPs, were reported using resequencing of wild populations (Das et al., 2020). Recently, high-quality *de novo* genome sequencing of rohu was carried out using next-generation sequencing platforms, resulting in 946 Mb-sized final genome and 2,844 unplaced scaffolds (Arick et al., 2023). Black carp’s genome survey assembly was performed by using Illumina sequencing technology. This centered on the evolution of genes or gene families linked to innate immunity as well as developmental distinctions from other carps (Lu et al., 2022). Recently, HiFi genome assembly of four major Asian domestic carps have been generated and available for various purposes including speciation, adaptation and evolutionary dynamic studies (Wang et al., 2024; Chen et al., 2024).

Complete mitochondrial genome sequencing of various carp species such as *L. catla* of 16,597 bp in length (Kibria et al., 2020) *L., rohita* of 16,606 bp in length (Das et al., 2020), *C. mrigala* of 16,594 bp in length (Bej et al., 2013) mud carp, *C. molitorella* of 16,602 bp in length (Zhang et al., 2015), *C. idella* of 16,609 bp in length (Wang et al., 2008a), black carp, *M. piceus* of 16,581 bp in length (Lin et al., 2016), and *L. fimbriatus* of 16,614 bp in length (Sahoo et al., 2016) has been reported. Mitochondrial DNA (mtDNA) finds extensive applications in conservation genetics and phylogenetic analysis of numerous freshwater fish species. Its high mutation rate makes it a valuable tool for detecting evolution and species boundaries (Das et al., 2013). In population genetics, mtDNA has proven to be useful for assessing genetic diversity, population structure, species identification, evolutionary studies, and estimating introgression from restocking efforts (Wenne, 2023a) as listed in Table 1. The most widely employed molecular method for species identification is DNA barcoding, which involves sequencing, amplifying, and analyzing specific regions such as the Cytochrome C oxidase 1 (COI) gene of the mitochondrial genome (Hallerman, 2021a).

**TABLE 1:** Genome sequencing in cyprinid species.

Species	Chromosome (2n)	Contig N50 (kb)	Scaffold N50 (Mb)	Scaffolds	Platform	Size (Mb)	Year	References
Common carp *(Cyprinus carpio)*	100	68.4	1.0	2,503	Roche 454, Illumina and SOLiD	1830	2014	Xu et al. (2014b)
	100	1,550	—	1,530	Illumina platform with 150-bp PE mode	1,680	2021	Li et al. (2021)
Black carp *(Mylopharyngodon piceus)*	48	0.6	0.0095	103,140	Illumina HiSeq _Ten platform	957	2022	Lu et al. (2022)
Big head carp *(Hypophthalmichthys nobilis)*	48	4.0	0.08	661,239	Illumina HiSeq 2000; PacBio	1,080	2020	Wang et al. (2020)
		39.1	3.33	353	Illumina HiSeq4000	844	2021	Jian et al. (2021)
		24.25	33.7	32	Illumina HiSeq; Oxford Nanopore PromethION	857	2021	Fu et al. (2021)
Grass carp (*Ctenopharyngodon idellus)*	48	40.7	6.5	301	Illumina HiSeq 2000	900	2015	Wang et al. (2015)
		19.3	35.7	30	PacBio (Sequel)	893.2	2022	Wu et al. (2022a)
Silver carp *(Hypothalmichthys molitrix*)	48	2.0	3.14	419,157	Illumina HiSeq 2000; PacBio	1,146	2020	Wang et al. (2020)
		19.9	0.9	861	Illumina HiSeq2000	782	2021	Jian et al. (2021)
Rohu (*Labeo rohita)*	50	30.6	1.95	3,623	Roche 454 (GS FLX), Illumina (Miseq and Nextseq500), Ion Torrent (PGM), and PacBio (Sequel)	1,480	2020	Das et al. (2020)
Catla (*Labeo catla)*	50	719.2	0.719	5,345	Illumina NextSeq; Oxford Nanopore MinION	1,000	2020	Sahoo et al. (2020)

Over the last decade, significant efforts have been devoted to sequencing and re-sequencing genomes of various carp species, employing multiple sequencing platforms and computational tools. Integration of transcriptomics, epigenomics, and other functional genomics techniques with whole-genome sequencing (WGS), researchers have made significant progress in identifying and characterizing genes linked to specific traits (Misra et al., 2019). However, the potential of this genomic information has not been harnessed for genomic selection for production enhancement or fisheries management.

### 4.2 Transcriptomics studies in cyprinid species

The transcriptome represents the complete set of genes in particular cells, tissues, or organisms at specific physiological conditions, developmental stages, and external environmental stimuli (Wang et al., 2009; Lindberg and Lundeberg, 2010; Heras, 2021; Qian et al., 2014). With the rapid development in the NGS and computational tools, several transcriptome studies in model and non-model fish were performed (Chandhini and Rejish Kumar, 2019). The transcriptomic analysis enables a systematic understanding of the gene profile and their expression patterns in fish (Barange et al., 2018; Chandhini and Rejish Kumar, 2019). RNA-seq approach is immensely used for analyzing dynamic transcriptome in non-model fish including carp species where genomic resources are not available (Qian et al., 2014). Numerous biological processes in carp species, including immunological response, biotic and abiotic stressors, developmental biology, etc., have been uncovered through RNA-seq. Fish-T1K project initiated by Beijing Genomics Institute, China, in November 2013 in collaboration with several research institutes, aimed at generating 1,000 transcriptome sequences of diverse fishes using RNA-seq approach (Sun et al., 2016). Under this initiative, transcriptome data of more than 200 species have been completed and the majority of species belonged to three orders such as Cypriniformes, Perciformes, and Cyprinodontiformes. Studies at the transcriptome level can assist in locating important loci or biomarkers to understand their function in physiological processes and reveal the way they respond to fluctuating environmental factors, including hypoxia, pollution, temperature, and salinity. We have given brief details of transcriptome studies undertaken in carp species in Table 2.

**TABLE 2:** Transcriptomic studies in cyprinid species.

Sr. No	Species	Tissue	NGS platform used	No of DEGs identified	Environmental variable/challenge/purpose	Key findings	Reference
1.	Common carp (*Cyprinus carpio*)	Brain	Illumina HiSeq 4,000	366 genes upregulated; 688 genes downregulated	Toxicity (Mercury)	Provides an insightful view of the toxic effects of mercury on brain injury of common carp	Zhang et al. (2023b)
		Brain	Illumina HiSeq 4,000	502 genes upregulated; 1,639 genes downregulated	Toxicity (Lead)	Explained the relationship between lead exposure and brain injury in common carp.	Zhang et al. (2020b)
		Brain	Illumina HiSeq 4,000	444 genes upregulated; 742 genes downregulated	Fluorine exposure	Provide insights into the mechanisms underlying brain injury induced by fluorine exposure.	Zhang et al. (2020c)
		Spleen	Illumina HiSeq2500	55 upregulated 14 downregulated genes	CyHV-3 virus infection	Identified genes and pathways that take part in disease resistance mechanisms in fish	Tadmor-Levi et al. (2019)
		19 different tissues	Illumina HiSeq 2,500	—	F1 hybrid strains	Identified sets of genes that are potential selective markers for various types of tissues	Kolder et al. (2016)
		Liver	Illumina HiSeq 4,000	444 genes upregulated; 338 genes downregulated	Injection of Insulin	Characterized the profile of genes expression response to insulin in common carp liver for the first time	Zhou et al. (2016)
		Liver	Illumina HiSeq 4,000	—	Hormone stimulation (GH)	It provides Growth Hormone (GH) affects various physiological activities by regulating gene expression	Zhong et al. (2016a)
2.	Songpu mirror carp (*Cyprinus carpio*) and Barbless carp (*Cyprinus pellegrini*)	Larvae	—	502 upregulated 1,061 downregulated	Low temperature	This data reveals insights into carp cold tolerance mechanisms.	Ge et al. (2020)
3.	Koi carp (*Cyprinus carpio*)	Muscle, Spleen	Illumina HiSeq 2000	1,001 upregulated 470 downregulated	*Aeromonas sobria* infection	Identified underlying immune mechanisms elicited during bacterial infection	Byadgi et al. (2018)
4.	Yellow River carp (*Cyprinus carpio*)	Ovary	Illumina Hiseq 2,500	19728 genes upregulated; 9,951 genes downregulated	Stages of ovary development	It contributes to the knowledge of ovary differentiation of Yellow River carp	Jia et al. (2018)
5.	Grass carp (*Ctenopharyngodon Idella*)	Muscle	Illumina HiSeq 2000	71 upregulated; 35 downregulated genes	Fast-growing and Slow-growing families	Biomarkers of growth in selective breeding programs for grass carp	Lu et al. (2020)
		Brain, Liver	Illumina HiSeq 4,000	1,622 DEGs (Brain) 2,534 DEGs (Liver)	Temperature	Revealed molecular regulation of heat stress in grass carp	Zhang et al. (2022)
		Intestine	Illumina HiSeq 2000	315 genes upregulated; 234 genes downregulated	Infection with bacteria (*A. hydrophila*)	Provides intestine-specific transcriptome data, allowing us to unravel the mechanisms of intestinal inflammation triggered by bacterial pathogens	Song et al. (2017)
		Gill, Intestine, Liver, Spleen	Illumina HiSeq 2000	—	Infection with virus (reovirus)	Results showed that immune responses occurred in all four tissues, indicating that GCRV probably does not target any tissue specifically.	Shi et al. (2014)
6.	Bighead carp (*Hypophthalmichthys nobilis*)	Palatal organ	—	—	Growth rate at different periods	Understanding functions and development mechanisms of palatal organ, and potential candidate genes that may be related to the genetic modulation of head size of bighead carp.	Wang et al. (2023)
		Head	Illumina Hiseq 2,500	Dph3 vs Dph1 (112 upregulated and 83 downregulated), Dph5 vs Dph3 (20 upregulated and 0 downregulated), Dph15 vs Dph5 (68 upregulated and 32 downregulated) and Dph30 vs Dph15 (35 upregulated and 50 downregulated)	At different developmental stages (Dph - Days post hatch)	It provides potential candidate targets for interaction regulation during early growth in bighead carp.	Luo et al. (2022)
		Bone	PacBio Iso-Seq	Parietal bone tissues (15 up- and 12 downregulated) 45 from vertebra tissues (24 up- and 21 downregulated)	Bone development	Developed novel full-length transcriptome resource	Luo et al. (2021)
7.	Silver carp (*Hypophthalmichthys molitrix*)	Liver	—	86 genes upregulated 98 genes downregulated	Toxicity (Microcystin-LR)	Revealed toxic effects of MC-LR and the antitoxic mechanisms of MC-LR in fish	Qu et al. (2018)
		Liver	Illumina HiSeq. 4,000	82 genes upregulated; 61 genes downregulated	Toxicity (*Microcystis aeruginosa*)	Few genes play major roles in the toxic, detoxifying, and antitoxic mechanisms of microcystin in fish	Hu et al. (2017)
8.	Black carp (*Mylopharyngodon piceus*)	Spleen	Illumina HiSeq X Ten	1,312 genes upregulated 623 genes downregulated	Disease resistance	It was observed that spleen of the infected group had necrosis	Zhang et al. (2018)
		Brain, Liver	Illumina HiSeq X Ten	Brain (630 genes upregulated and 627 genes downregulated) Liver (6,540 genes upregulated and 5,940 genes downregulated)	Fasting	Provided molecular response mechanism of black carp under fasting conditions	Dai et al. (2021)
		Liver, Muscle	Illumina HiSeq X Ten Platform	834 genes upregulated and 1,079 genes were downregulated	Different growth rates	DEGs obtained help in understanding the growth traits of the black carp	Zhang et al. (2020a)
9.	Rohu (*Labeo rohita*)	Muscle	Illumina Miseq platform	15 genes upregulated and genes 37 downregulated	Thermal stress	Understanding of stress-responsive biomarkers due to thermal adaptations in farmed carps.	Kumar et al. (2023)
		Liver, muscle	Roche 454 GS-FLX next-generation sequencing platform	—	Selective breeding	Marker resources developed would support the genetic improvement program of rohu.	Sahoo et al. (2022)
		Head kidney	Illumina Hiseq 2,500	—	Infection with *Aphanomyces invadens*	Understanding of the host-pathogen interaction that might underpin the development of new management strategies for this economically devastating fish-pathogenic oomycete *A. invadans.*	Pradhan et al. (2020)
		Liver	Illumina Hiseq 500	40% carbohydrate diet (4,464 genes upregulated; 4,343 genes downregulated) 60% carbohydrate diet (4,478 genes upregulated; 4,171 genes downregulated)	Different dose of Carbohydrate in the diet	Exploration of early nutritional programming for enhancing glucose efficiency in carp species, for sustainable and cost-effective aquaculture production.	Rasal et al. (2020)
10.	Gold fish (*Carassius auratus*)	Embryo	Illumina HiSeq 2000	—	Artificial embryo selection	Artificial selection has led to far-reaching influences on goldfish gene expression	Du et al. (2023)
		Gills	Illumina Hiseq 2000	1829 genes upregulated; 2013 genes downregulated	Infection with bacteria (*Aeromonas hydrophila*)	Understanding on the molecular mechanisms of local mucosal immunity in cyprinid species	Hu et al. (2023)
		Skin	Illumina HiSeqX	344 genes upregulated; 212 genes downregulated.	Infection with parasite (*Gyrodactylus kobayashii*)	It provides better understandings of immune defense mechanisms of goldfish against *G. kobayashii.*	Zhou et al. (2021a)
		Brain	Illumina Hiseq 2000	132 genes upregulated; 114 genes downregulated.	Feed Conversion Efficiency (FCE)	Provides useful pathway information and candidate genes for future studies of genetic mechanisms underlying FCE in crucian carp.	Pang et al. (2018)

The first transcriptome sequencing in common carp to identify innate immune genes responding to *Mycobacterium marinum* discovered 39 TIR (Toll/interleukin-1 receptor) domain-containing transcripts/genes (Zhang et al., 2011). Similarly, in rohu, transcriptome sequencing revealed several transcripts associated with the immune response to *A. hydrophila* challenge, including *HSP70, HSP90, HSP30*, major histocompatibility (*MH*), and *glycoproteins* or *serum lectin* (Robinson et al., 2012). In the last 5–6 years, there has been significant progress in transcriptome work in carps, particularly focusing on metabolism, growth, disease, biotic or abiotic stress, immunity, and related areas.

In black carp, transcriptome sequencing of liver and brain tissues revealed 13,737 differentially expressed genes (DEGs) in response to fasting (Dai et al., 2021). In grass carp, liver and brain transcriptome analysis under heat stress (36°C) vs. control (28°C) exposure identified 2,534 DEGs in the liver and 1,622 in brain tissue (Zhang et al., 2022). A liver transcriptome analysis in genetically selected Jayanti rohu (*L. rohita*) revealed 15,232 and 15,360 differently expressed transcripts when fed with a high carbohydrate diet (40% and 60% starch level) as compared to the control diet (20% starch level), demonstrating upregulation of critical transcripts related to glycogen production and *de novo* lipogenesis (Rasal et al., 2020). “FishGET,” a recent development, offers a comprehensive gene expression and transcriptome database for eight species, including grass carp. It facilitates the discovery of new genes and non-coding RNAs, serving as a valuable reference for transcriptome annotations (Guo et al., 2023). The “FishGET” RNA-seq database contains 1,362 paired-end data from 97 bio-projects across 8 species, providing extensive sequence data for getting valuable insights into various aspects of carp biology, such as growth, reproduction, nutrition, stress response, adaptation, and immunity.

### 4.3 Epigenetic factors (DNA methylation and miRNAs) in cyprinid species

Epigenetics is the study of changes in the expression of genes and/or phenotypes of organisms that occur without a change in the underlying DNA sequence (Gibney and Nolan, 2010; Liu et al., 2022). Earlier, epigenetic research in aquaculture species was often ignored, but in recent years it has drawn attention to the biological responses of fish to stress, pollution, disease, and food (Metzger and Schulte, 2016; Gavery and Roberts, 2017; Rasal et al., 2023). The epigenetic alterations of DNA methylation, histone modification, and micro RNAs (miRNAs) are significant in a variety of biological processes, including development, reproduction, and growth (Feil and Fraga, 2012; Xu and Xie, 2018). DNA methyltransferases (DNMT) are involved in the addition of a methyl group to the cytosine base during DNA methylation (Luo et al., 2018). Methylation of CpG sites in gene coding regions or promoter sequences can inhibit transcription, while intragenic methylation may stimulate transcription (Jones et al., 2015; Han et al., 2022). The accessibility of fish whole genome sequences enables the identification of DNA methylation sites.

Previous studies suggested that epigenetic factors might be included in selective breeding programs since they operate as a mediator between phenotype and genotype (Anastasiadi et al., 2021; Granada et al., 2018). During the last 3–4 years, epigenetic research has been concentrated on carp species in response to environmental conditions or metabolic stressors, disease, etc. Recently, great efforts have been made to decipher epigenetic regulation in disease control in candidate aquaculture fish, including carp. In grass carp, genome-wide DNA methylation studies depicted that age-dependent epigenetic factors play a crucial role in grass carp reovirus (GCRV) susceptibility (He et al., 2022). Using the whole-genome bisulfite sequencing (WGBS) approach, this study identified 6,214 differentially methylated regions (DMRs) and 4,052 differentially methylated genes in five-month-old (FMO) and resistant three-year-old (TYO) grass carp, respectively. This work suggests the role of epigenetics in gene regulation, which is influenced by the age of fish during viral disease infection, and that can be helpful in the development of breeding programs for disease-resistance traits. In common carp, methylation studies at the global and gene-specific levels in fresh and old spermatozoa reveal that sperm aging impacts the effectiveness and rate of fertilization (Cheng et al., 2021). Similarly, increased H4K12ac expression level was found to be related to aging oocytes in common carp (Waghmare et al., 2021).

Studies on DNA methylation in Crisp grass carp (CGC) and improved varieties of grass carp (GC) have shown that faba bean (*Vicia faba* L.) diet-induced epigenetic alterations contribute to the development of muscle (Li et al., 2022). This work identified a total of 14 key differentially methylated genes associated with muscle development and provided insights for nutritional programming research. Studies have shown that the growth feature is crucial for aquaculture output and that allotriploid carp heterosis and growth are regulated by DNA methylation (Ren et al., 2022). In Supplementary Table S1, we have compiled a summary of DNA methylation studies conducted in carp species.

MicroRNAs are a type of epigenetic factor, non-coding single-stranded (18–23 nt) small RNA involved in the regulation of gene expression during cell development and other biological events (Rasal et al., 2016). These microRNAs predominantly bind to the 3′untranslated region (UTR) of target genes, leading to the repression or degradation of their target mRNA, thereby facilitating regulatory mechanisms. In teleost, numerous miRNAs have been identified and documented in the miRNA database (http://www.mirbase.org/), with 280 miRNA entries reported for the common carp. Similarly, in carp species, the presence of miRNAs has been reported in response to several biological events and depicted their regulatory role. Earlier studies in carps revealed that environmental or metabolic factors may influence the transcriptional processes via mRNA-miRNAs interactions. Based on the rapid refeeding response after fasting in grass carp, eight miRNAs (miRNA-1a, miRNA-133b, miRNA-133a, miRNA-181a, miRNA-214, miRNA-146, miRNA-206, and miRNA-26a) in fast skeletal muscle were identified, and expression analysis suggested their role during myogenesis (Zhu et al., 2014).

MicroRNA profiling in carp species provides insight into the role of miRNAs in numerous biological functions. Profiling of microRNAs in the liver of common carp identified five major miRNAs (miRNA-137, miRNA-143, miRNA-21, miRNA-146a, and miRNA-125b) to be related to oxidative stress in response to different diets (Yang et al., 2019). Similar to this, prolonged exposure to 1-methyl-3-octylimidazolium bromide (C8mimBr) affected the expression of miRNA-125b, miRNA-155, and miRNA-21 in the spleen of silver carp, which suggested their role during inflammation and oxidative stress (Ma et al., 2019). In *Labeo bata*, a total of 231 conserved and 445 novel liver-specific microRNAs linked to the carbohydrate metabolic pathway were identified (Rasal et al., 2019). Similarly, in rohu, miRNA studies identified 138 conserved and 161 novel liver-specific miRNAs associated with carbohydrate metabolism. Further expression analysis of five key miRNAs, namely miR-22, miR-122, miR-365, miR-200, and miR-146, suggested their involvement in metabolism when fed with high carbohydrate diets (Rasal et al., 2020). To provide a comprehensive overview of the miRNA studies conducted in carp species, we have compiled the information in the Supplementary Table S1.

MicroRNA studies in carp species are at an early stage and have emerged as a fundamental factor or biomarker for understanding their role in gene regulation. In response to various environmental stresses, diseases, and feeding habits, research on miRNA in a selected carps has considerably increased during the past several years. Their molecular interaction with the target genes must be defined in more detail to serve as a biomarker for the improvement of aquaculture practices.

### 4.4 Microbial characterization in cyprinid species

The term “metagenomics” refers to the study of the structure and function of entire nucleotide sequences isolated and analyzed from all the organisms (often but not always microbes) in a bulk sample (Thomas et al., 2012). This allows the scientific community to explore the diverse range of microbes that inhabit in mammals, oceans, soils, fishes, and the environment (Frank, 2008; Ashwath et al., 2022). Advances in DNA sequencing and computing methods have achieved paradigm shifts in metagenomics, enabling comprehensive study of the complex microbial system. Genomics analysis has undergone a significant shift towards metagenomics, allowing the study of entire microbial communities without the necessity of isolating and culturing individual species. Given the vast diversity of fishes in terms of species and habitats, it is crucial to conduct ecological assessments that identify microbes and their diversity both in the aquatic environment and within the fish gut. Fish gut microbiota is play an important role in the microbial fermentation of feed which adds to the host’s health (Flint et al., 2008; Round and Mazmanian, 2009; Wu et al., 2012; Ringø et al., 2003).

The diversity of microbes was studied based on the 16sRNA gene sequencing, which is highly diverse among microbes, but it does not provide a functional aspect (Vaishampayan et al., 2010; Johny et al., 2021). The bacterial diversity has been studied in grass carp, rohu, common carp, gibel carp, etc., by harboring 16sRNA sequencing (Mukherjee et al., 2020). In common carp, bacterial diversity was identified based on various aspects such as the effect of pollutants, bacterial or viral challenges, antibiotics, etc. As the skin-mucus bacterial diversity determines the health of the host, skin-mucus bacterial diversity in common carp was assessed and showed the presence of dominant phyla such as *Proteobacteria*, followed by *Actinobacteria*, *Bacteroidota*, *Firmicutes*, and *Cyanobacteria* (Papp et al., 2023). In grass carp, the majority of *Proteobacteria* was replaced by *Bacteroidetes* during larval feeding as opposed to the egg stage (Wang et al., 2015). Gut microbial diversity in silver carp demonstrated seasonal clustering (Ye et al., 2014). In goldfish *Bacteroidetes* were observed dominantly in response to pentachlorophenol (PCP) exposure (Kan et al., 2015). Studies suggest that the feeding behavior of various carp species has a notable impact on the composition of their gut microbiota (Li et al., 2017a). Studies carried out on grass carp revealed correlated gut microbiota composition following the regime (Ni et al., 2014). The bacterial diversity in the gut of fish influences fish physiology as well as health. In the crucian carp, opportunistic bacteria like *Vibrio*, *Aeromonas*, and *Shewanella* were found to be more during infection with the red-operculum disease (Li et al., 2017b). Similarly, changes in gut microbiota were seen in rohu in response to argulus infection, revealing the presence of *Actinobacteria*, *Patescibacteria, Stenotrophomonas* and *Pirellula* in infected fish (Mondal et al., 2022).

The chronic toxicity of Norfloxacin (NOR) concentrations on the gut microbiota of juvenile common carp was evaluated using 16sRNA sequencing. The prevalent bacteria in the gut of common carp included *Proteobacteria, Bacteroidetes, Fusobacteria, Firmicutes,* and *Actinobacteria* (Li et al., 2023)*.* Analyses of acquired antibiotic-resistance genes (ARGs) in the intestinal microbiome of common carp indicate a possible link between acquired ARGs in domestic and wild animal populations (Libisch et al., 2022). Meta-transcriptomic viral survey of invasive and native common carp across the Murray–Darling Basin did not detect CyHV-3 or any closely related viruses. This suggests that there is little virus transmission from common carp to native fish species (Costa et al., 2021). Herewith, we have summarized the identification of bacterial diversity in carp species using 16 S RNA sequencing approaches in Supplementary Table S2. Overall, 16sRNA sequencing approach in carp species revealed that bacteria in the fish gut microbiome are dominated by the phyla *Firmicutes, Bacteroidetes, Actinobacteria, Fusobacteria, Bacilli, Clostridia,* and *Verrucomicrobia* (Xia et al., 2014; Wu et al., 2012; Roeselers et al., 2011). While *Proteobacteria*, *Firmicutes*, and *Bacterioidetes* contribute around 90% of fish gut bacterial diversity which showed their physiological role during digestion, immunity, and nutrient acquisition (Ingerslev et al., 2014; Johny et al., 2021).

Hologenome concept views the holobiont comprising a host and its microbiomes as the primary unit of selection in evolution (Alberdi et al., 2022; Limborg et al., 2018). Earlier research underscores the critical role that microbiomes play in host fitness and local adaptation. Hologenomics studies, which examine the genetic interactions between hosts and their microbial communities, hold great promise but remain an underexplored field. This approach could significantly improve aquaculture by enhancing growth, health, and sustainability. For instance, studies on invasive carp species in the Mississippi River Basin suggest that hybrid carp gut microbiomes contribute to their successful adaptation and invasion by aiding in food resource digestion (Zhu et al., 2021). While hologenomics shows great potential, especially in aquaculture, it remains in its early stages and is not yet widely applied.

### 4.5 Proteomics and metabolomics in cyprinid biological research

A proteome is a representation of the whole protein complement of the genome. Proteome composition varies over time between tissues and is studied through proteomics, which is the study of all proteins in a certain cell, tissue, or organism (Rodrigues et al., 2018). Thus, although the genome is constant for organisms, proteome content is dynamic and changes as per the developmental stage or in response to external stimuli. The studies suggest that this field is useful for understanding fish developmental biology and could be useful for the identification of novel biomolecules or proteins. Several researchers attempted to review progress made in proteomics research in fisheries (Nissa et al., 2021; Jaiswal et al., 2023). In carp species, proteomic research is at an infancy level, and recently it gained attention to study proteome of biological systems in response to disease, stress, or physiological stages. In addition to these, this field is also being utilized for examining fish food quality and authentication using protein biomarkers (Mazzeo and Siciliano, 2016). Earlier studies showed that proteomic information could be used for toxicological research in carps, such as in goldfish (Wang et al., 2008b).

Mass spectrometry was used for the first time to analyze the spermatozoa of common carp, identifying 348 proteins, including numerous proteins such as Dynein, tubulin, HSP70, HSP90, HSP60, NKEF-B, adenosylhomocysteinase, brain type creatine kinase, mitochondrial ATP synthase, and valosin (Dietrich et al., 2014).

In cyprinid aquaculture, diseases are the major hurdle in production, and thus understanding fish immunity and host-parasite relationship could be helpful to devise a strategy for disease control. The proteomic study of host tissues linked to pathogenic infection is a powerful approach to identifying signature proteins as shown in common carp, grass carp, and rohu. In rohu, liver proteomic analysis revealed 158 differentially expressed proteins in response to *A. hydrophila* infection (Nissa et al., 2023). Similarly, in common carp, alteration of proteome was observed in skin mucus due to *Ichthyophthirius multifiliis* infection, and several key biomarker proteins were identified, such as dermatopontin, lumican, olfactomedin 4, papilin I, and cytoskeletal 18 (Saleh et al., 2018).

Despite these efforts, there is still a need to investigate carp fish proteomes in response to environmental stressors, pollutants, pathogens, etc. The research communities need to have access to a comprehensive proteome of significant carp species. Recently, an open-source database named “Rohu PeptideAtlas” has been released. This resource contains 6,015 proteins, 2.9 million PSMs (Peptide-Spectrum Matches), and approximately 150 thousand peptides sourced from 17 distinct normal tissues, plasma, and embryos of rohu. This valuable repository will play a crucial role in supporting the carp aquaculture communities (Nissa et al., 2022).

Metabolomics employs high-throughput mass spectrometry (MS) techniques to analyze and estimate the content of metabolites, with a molecular weight below 1,500 Da, within an organism (Alfaro and Young, 2018; Kumari et al., 2022). Earlier, those sorts of studies were applied in humans, but recently metabolomics studies have gained consideration in animals, including fishes for environmental, health, and nutritional research (Brennan, 2013; Goldansaz et al., 2017; Roques et al., 2020). In fish nutritional aspects, metabolomic provides insights of metabolic effects in response to different feed and nutrient content as reviewed earlier (Kumari et al., 2022). In metabolomic studies, several compounds such as carbohydrates, fatty acids, amino acids, nucleotides, steroids, organic acids, and carotenoids can be studied. In a holistic approach, this field helps to identify the complexity of fish species in response to nutrition and environmental conditions (Sébédio, 2017; Young and Benton, 2018).

In cyprinid species, metabolomic research is very limited, and recently few studies depicted its use in understanding the impact on fish nutrition and health (Alfaro and Young, 2018). When fed, The metabolic profile of grass carp in response to imbalanced diet with low levels of protein showed a change in lipid content with SFA (Saturated Fatty Acids), MUFA (Mono Unsaturated Fatty Acids), and PUFA (Poly Unsaturated Fatty Acids owing to regulation of choline and end products of glycerol and glycerol-3-phosphate (Jin et al., 2015b). Thus, diet plays a significant role in the metabolic profile of fish. In grass carp, metabolomic studies revealed dietary effects on flesh quality when fed with grass and artificial feeding. While grass-fed animals had greater levels of n-3 unsaturated fatty acids (UFAs) such as eicosapentaenoic acid, alpha-linolenic acid, and gamma-linolenic acid, those fed artificially had higher levels of arachidonic acid, docosapentaenoic acid, and gamma-linolenic acid (Zhao et al., 2018).

It was suggested that environmental conditions, pollutants, and abiotic factors could also affect the health/welfare and metabolism of fish. To understand the physiological mechanism of anoxia in crucian carp, the metabolic profile investigated high levels of succinate in different tissues, including brain, liver, heart, and blood plasma, as compared to normoxic (control) fish (Dahl et al., 2021). Recently, in Qingtian paddy field carp (*C. carpio* var *qingtianensis*), metabolite profile in response to hypoxia revealed 131 differentially expressed metabolites as compared to control groups, and a total of 63 metabolites with differential expression were noticed in reoxygenated and the hypoxic fish (Jiang et al., 2022b). The impact of toxicant such as endosulfan sulfate was studied on dead or live common carp using GC–MS-based metabolome profiling and a total of 30 significant metabolites (biomarkers) associated with starch, galactose, glycerolipid, and sucrose metabolism were identified among dead and live carp (Lee et al., 2020). Similarly, metabolomics explored the harmful effects of 17-Ethynylestradiol (EE2) in crucian carp. This led to the identification of 24 metabolites in the gonad and 16 metabolites in the kidney, as well as the disruption of lipid metabolism. A specialized database (reference spectra of the metabolites) is still needed for fish metabolomics investigations, particularly in carp species, to examine inter- and intra-species metabolome profiles (Goldansaz et al., 2017).

## 5 Molecular maker discovery in cyprinid species

Molecular markers are DNA sequences in the gene or genome that show genetic alterations at a particular location leading to genetic variation. This depicts the differentiation in the form of DNA polymorphism between organisms, species, population genetics studies at population levels that can be used for mapping and identifying individuals. Several authors reviewed the progress made in molecular marker discovery and their applications in the teleost species (Chauhan and Rajiv, 2010; Wenne, 2023a). In carp species, several genetic markers were identified for assessment of genetic diversity, parentage assignments, species detection, population structure analysis, evolutionary study, linkage map construction, and selective breeding. It includes RAPD, RFLP, AFLP, microsatellites (SSRs), ESTs,SNPs, etc. Genetic markers also have been used for the conservation of genetic resources, stock differentiation, finding quantitative trait loci (QTL), and fisheries management. The use of proteins and allozymes as markers arose in the 1960s, but since has declined.

However, it has been observed that most of these phenotypic and biochemical markers exhibit limited polymorphism when studied in the majority of carp strains (Crooijmans et al., 1997), which restricted its application. RAPD and AFLP have limited applicability due to their drawbacks, including dominant nature and irreproducible findings, respectively. On the other hand, genetic markers such as microsatellites, single nucleotide polymorphisms (SNPs), expressed sequence tags (ESTs), and mitochondrial (mtDNA) markers have become prevalent in aquaculture-related research. These new markers offer more reliable and diverse options for studying genetic variations in aquatic species. The use of molecular markers has augmented classical phenotype-based selection. These molecular markers increase accuracy in identification of superior individuals or families. Although, several studies implicated the use of markers in the cyprinid aquaculture and management, recently developed NGS and computational tools have enabled precise DNA marker discovery associated with traits of interest (Sundaray et al., 2016; Rasal et al., 2017b). This section aims to provide an overview of the most recent applications of genetic markers in studying cyprinid species, which consistently rank as the top performers in Indian aquaculture productivity.

Microsatellites have found extensive use in research focusing on fisheries and aquaculture since the early 1990s. The microsatellite also known as simple sequence repeats (SSRs) are highly polymorphic DNA markers, which are neutral, co-dominant, high polymorphism, consisting of short 2-4 bp nucleotide repeats and wide distribution in organisms’ genomes (Sundaray et al., 2016; Wenne, 2023a). In earlier studies, they have been widely used for population genetic structure analysis, QTL mapping, stock characterization, marker-assisted selection, and breeding. The next-generation sequencing (NGS) genome assay identifies multiple microsatellites, of which a few highly variable loci serve adequate for population genetics and aquaculture applications (Mojekwu and Anumudu, 2013).

A total of 17 microsatellite loci showed 86.0%–96.0% genetic identity during the genetic differentiation among hatchery and wild populations of Oujiang common carp, *C. carpio* var. *color* (Ma et al., 2011). Similarly, 10 microsatellite loci were used during the analysis of population structure and genetic diversity of 11 carp strains from Germany (Scheuerman and Glinzig mirror carp), France (Forez and Dombez scaly carp), Amur river farmed stock, and one wild stock from the Ebro river (Spain), and it demonstrated 89.6% accuracy bayesian clustering (Ma et al., 2011). To analyze population structure and genetic diversity in silver carp, a total of 159 SSRs (27 polymorphic loci) were identified using transcriptome sequences, and a total of 134 SSRs (65 loci being polymorphic) markers were developed using wild silver carp populations (Feng et al., 2014; Guo et al., 2013).

Recently, SSRs were generated using the genome of silver carp to assess the genetic variation of the four populations, and they found a total of 368,572 SSRs or 0.77% of the genome. The most prevalent SSRs were discovered to be di-nucleotide repeats (55.59%) with AC/GT, and 13 pairs of SSRs were used to illustrate the genetic variation across four groups (Wang et al., 2022c). Similarly, in common carp, analysis of whole genome shotgun (WGS) sequences revealed a total of 79,014 microsatellite loci, and genotyping with 192 individuals showed 963 polymorphic markers that could be used for high-density linkage mapping (Ji et al., 2012). To evaluate genetic purity and genetic diversity of the 13 strains of Hungarian common carp, 12 SSRs markers were used and showed a significant level of genetic differentiation (3.79%) between strains with 93.64% accuracy in bayesian clustering analysis (Tóth et al., 2020).

Initially, 21 SSRs were identified for population structure analysis in rohu (Patel et al., 2008). Subsequently, an extensive investigation of rohu transcriptome data revealed a total of 22,383 EST-SSRs, with 29 of them significantly associated with reproduction-related transcripts (Sahoo et al., 2016). FishMicrosat: a microsatellite database was developed for various fishes, which includes important carp species such as *L. rohita, L. catla, C. idella,* and *H. molitrix* (Nagpure et al., 2013). This database serves as an important resource of microsatellite markers for evolutionary, population structure, cross-species loci identification, and genetic relatedness among carp species. Microsatellite markers were used to examine genetic variation among nine catla strains before the start of a selective breeding program. The study found a genetic heterogeneity with 0.4137 F_ST_ estimates, and 58.63% of the variation was observed among the individuals (Mahapatra et al., 2018). These markers were successfully used for base population establishment during the catla selective breeding program. Several SSRs in black carp were found, and their application in genetic diversity analysis across wild and farmed populations was demonstrated (Zhou et al., 2020). We have summarized the identification of SSRs markers in carp species with their uses in Supplementary Table S3.

High abundance SNP markers is a popular choice for population genetic studies in aquaculture (Wenne, 2023b; Abdelrahman et al., 2017; Rasal et al., 2017b). Millions of SNPs have been identified in carp species due to advancements in NGS technology, as shown in Supplementary Table S3. These SNPs are useful in generating linkage maps, identifying key phenotypic QTLs, and marker-assisted selection (MAS) in aquaculture (Yáñez et al., 2016). It is possible to decode connections between SNP markers and production or performance attributes using genome-wide association studies (GWAS) (Robledo et al., 2018). For the analysis of 68 individuals from second-generation mirror carp hybrids, *C. carpio*, 336 SNPs were taken into account (Jin et al., 2012), and further studies implicated that a total of 978 SSRs could be used for genetic map construction, consisting of 50 linkage groups (Jin et al., 2015a). A genomic map of the common carp was created utilizing 8,487 SNPs spanning 3762.88 cM and 50 linkage groups (Laghari et al., 2014; Xu et al., 2014a; Liu et al., 2017).

Carp genome re-sequencing reveals millions of SNPs associated with growth or disease resistance. In rohu, through whole genome sequencing and re-sequencing of 10 wild populations, 4.95 million SNPs (380,991 to 679,963) were identified in each population (Das et al., 2020). A total of 9,157 SNPs were found in rohu breeding populations from Halda, Jamuna, and Padma rivers. Among them, 1985 SNPs exhibited slight genetic divergence between the river populations (Hamilton et al., 2019b). The rohu genome and transcriptome resequencing revealed 39,158 high-quality coding SNPs. Twelve of these SNPs were linked to genes involved in cellular development and proliferation, including *myf 6, fgf, gfr*-*bound protein 10-like*, *IGF-1-like,* and *TGF β*, among others (Nandanpawar et al., 2023). In grass carp, two SNPs were found to be associated with increased resistance to reovirus and *tlr7* expression (Su et al., 2018). Recent resequencing of grass carp genomes identified a total of 9,510,648 SNPs across three populations: Yangtze, Pearl river, and mono-female population, indicating a moderate genetic differentiation between them (Zhang et al., 2023a). In common carp, a 250 k SNP array was developed using re-sequenced genome data, with 84,933 (34.0%) SNPs found to be polymorphic (Xu et al., 2014a). The performance of 24 morphological and growth-related parameters in yellow river carp was evaluated using this SNPs array. The gBLUP method showed medium to high heritabilities of these traits as compared to the traditional BLUP method, with an increase in prediction abilities by 2% (Jiang et al., 2022a).

## 6 Gene transfer in cyprinid species

Transgenesis, a revolutionary science, involves creating genetically modified organisms by integrating a foreign DNA sequence into its chromosomal DNA (Wang et al., 2021b). Various techniques, such as microinjection, electroporation, transposable elements, and retroviral-mediated transfer, have been employed to produce transgenic individuals. The primary objectives of genetic manipulation in aquaculture include intensifying growth and improving food conservation efficiency, enhancing tolerance to environmental variables such as temperature and salinity, introducing new color variants in ornamental fishes, and developing disease-resistant forms. Microinjection and electroporation have proven to be reliable techniques for gene transfer in fish, with goldfish being the first reported transgenic fish, followed by advancements in species like rainbow trout and common carp (Zhu et al., 1985). Growth hormone (GH) genes, known for their highly conserved sequences, have been a central focus in transgenic studies, targeting over 20 teleost species. Transgenic applications in ornamental fish, particularly those utilizing reporter genes like green fluorescent protein (GFP) or red fluorescent protein (RFP), have led to the commercialization of species like the “GloFish” series, featuring attractive fluorescent colors. While much of the transgenic research in food fish has centered around growth-related traits, other aspects such as disease resistance, tolerance to environmental conditions, and improvements in feed conversion ratio have been explored. The AquAdvantage Salmon, a growth-enhanced transgenic food fish, stands out as the first transgenic fish commercially approved by the US Food and Drug Administration (FDA).

The journey into transgenic fish production in carps began in the mid-1980s when researchers achieved a breakthrough by integrating recombinant human growth hormone (rGH) into red common carp embryos (Wu et al., 2003). These transgenic fish exhibited a remarkable growth rate, surpassing four times that of the control group, showcasing the potential and efficiency of gene transfer in fish breeding. The incorporation of specific promoters, such as the *β-actin* promoter from carp, facilitated the construction of all-fish transgenic constructs. The “auto transgenesis” approach, utilizing regulatory elements and targeted genes from the same species, proved beneficial in transgenesis. This has led to the development of an “all-fish (CAgcGH, grass carp *growth hormone* fused with common carp *β-actin* promoter)” transgenic yellow river carp, where the GH from grass carp was introduced, adhering to biosafety and bioethics standards (Wu et al., 2003). This transgenic common carp not only demonstrated faster growth and enhanced food conversion efficiency but also addressed environmental concerns by producing infertile transgenic triploid fish. These findings were further expanded the scope by introducing the GH of black carp into hybrids of *C. auratus red* var. and *C. carpio*, which exhibited rapid growth. Similarly, researchers enhanced the growth of rohu using an “all-fish” transgene construct (GH cDNA from rohu attached to a *β-actin* promoter from grass carp), resulting in transgenic individuals exhibiting 5–6 times greater growth than their non-transgenic counterparts (Devlin et al., 2009; Rajesh and Majumdar, 2005). The *β-actin* promoter, showing variable but sometimes ubiquitous expression, was studied in medaka (Cho et al., 2011). The transgenesis using rohu *β-actin* gene and promoter, demonstrated the potential of endogenous promoters in driving foreign gene expression (Barman et al., 2017). Further studies, *mylz2* promoter of rohu carp (1.2 kb) was successfully cloned and direct injection into skeletal muscle showcased the potential for tissue-specific and desired gene expression in transgenic rohu (Barman et al., 2017).

In common carp, other traits such as feed conversion efficiency, appetite, and elevated post-feeding routine oxygen consumption rates are being explored. Transgenic studies extended to the manipulation of fatty acid profiles, as demonstrated by the successful generation of *fat1* and *fat2* single- and double-transgenic EPC cell lines (Zhang et al., 2019b). These studies resulted in “all-fish” fat1-transgenic common carp populations with significantly increased n-3 PUFA content, emphasizing the potential of transgenic technology to enhance the production of essential fatty acids for human consumption. Moreover, exploring genetically modified fish as bioreactors for producing target proteins is gaining attention. For instance, transgenic zebrafish expressing the human fat1 gene have successfully produced omega-3 long-chain polyunsaturated fatty acids (n-3 LCPUFA), crucial for neural development and human health (Pang et al., 2014). The optimization of Tg (CA: fat1) common carp, achieved through a sperm sample screening method, holds promise as a novel dietary source of omega-3 polyunsaturated fatty acids (n3-PUFAs) (Zhang et al., 2019b). Dietary protein effects on growth and feed utilization were studied in GH transgenic and non-transgenic common carp (Guo et al., 2021). Higher protein improved growth, reduced body lipid, and enhanced metabolic processes in transgenic fish, indicating better overall performance compared to non-transgenic counterparts. Recent studies, GH transgenic common carp demonstrated notable aquaculture benefits, boasting a 12% higher specific growth rate (SGR) and 17% higher feed efficiency (FE) compared to their wild-type counterparts. This investigation explores lipid metabolism in GH transgenic and wild-type common carp, uncovering diminished lipid content across diverse tissues in GH transgenic carp (Wu et al., 2022b). The findings showcase enhanced lipid metabolism and utilization pathways in their liver, providing valuable insights for optimizing lipid efficiency in aquaculture. Interestingly, studies also reported enhancement of food intake GH common carp mediated by upregulating the hypothalamic Agouti-related protein (AgRP) (Zhong et al., 2013).

Transgenic fish with enhanced disease resistance contribute to increased aquaculture production and improved fish welfare (Dunham, 2009). Efforts have been made in this direction by incorporating genes for bactericidal compounds, such as cecropin in channel catfish and human lactoferrin (hLF) in grass carp, to bolster the nonspecific disease resistance of cultured fishes (Wang et al., 2022a). One notable example is the transfer of the hLF gene, creating transgenic grass carp with resistance to *Aeromonas hydrophila* (Wang et al., 2021b).

The development of transgenic fish has witnessed significant progress in carps, addressing not only growth-related traits but also essential fatty acid composition and tissue-specific gene expression. However, transgenic fish commercialization is limited due to ecological concerns and social issues. To mitigate ecological risks, creating sterile transgenic fish has been explored through ploidy manipulation or hormonal treatment. Confinement strategies, both biological and physical, are proposed to address the potential escape of transgenic fish into the wild. Although several transgenic food fish have been developed, only the AquAdvantage Salmon has been globally commercialized, mainly due to societal and ecological considerations. Biosafety assessments are crucial, and adherence to international guidelines is necessary for the responsible development and deployment of transgenic fish technologies. The challenges include achieving stable transgene integration, mosaicism, and optimizing regulations for safe laboratory practices and biosafety. The integration of traditional selective breeding and transgenic approaches may be essential to meet the growing demands for future food requirements.

## 7 Genome editing in cyprinid species

Genome editing also known as gene editing is a method that involves making specific changes to the DNA or region of the genome of a cell or organism. Genome editing technologies like transcriptional activator-like effector nucleases (TALENs) and zinc finger nuclease (ZFN) were early methods for targeted genome editing (Cong et al., 2013; Beerli and Barbas, 2002). However, they have been largely replaced by the more efficient and versatile CRISPR/Cas9 system i.e., CRISPR (clustered regulatory interspaced short palindromic repeats)/CRISPR associated nucleases (Cong et al., 2013). CRISPR/Cas9 allows for precise editing of genomes, including gene knockout, activation, inhibition, and even epigenetic modifications, revolutionizing biological research and offering new possibilities for understanding and modifying DNA. Several authors have reviewed the progress of genome editing in aquaculture species and explored new possibilities for enhancing genetic improvements in cultured aquatic species (Hallerman, 2021b; Barman et al., 2017; Gratacap et al., 2019; Gutási et al., 2023; Ferdous et al., 2022). This signifies the potential for cutting-edge genetic technologies to advance aquaculture and improve the quality, growth, and sustainability of farmed aquatic species.

Selective breeding in cultured species is constrained by factors like low heritability of traits, long generation-intervals, the complexity of targeting multiple traits, and limited genetic variation in broodstock. CRISPR/Cas9 offers the potential for rapid genetic improvement in aquaculture by targeting specific traits like sterility, growth, and disease resistance. CRISPR/Cas9 and TALEN genome editing techniques have been effectively used in several aquaculture species, including cyprinids such as common carp, rohu, and grass carp.

In common carp, genome editing tools were used to target genes related to bone development (*SP7, SPP1, RUNX2,* and *OPG*) and muscle growth (*Myostatin*) (Zhong et al., 2016b). TALEN induced mutations in specific target sites, while CRISPR-Cas9 caused severe bone-defects when targeting *SP7* genes and increased muscle growth when targeting *MSTNba*. Moreover, CRISPR-Cas9 enabled the creation of double mutant fish with high efficiency (*SP7A; MSTNba*). These technologies in common carp represent a significant first step forward in genetic engineering for cyprinid species, with the potential to enhance their qualities and economic value in aquaculture. Recently, CRISPR/Cas9 was used to knockout the *MSTN* gene in common carp, resulting in fish with increased muscle growth (Shahi et al., 2022). This study was supported by the observed increase in skeletal muscle fiber-density and the upregulation of myogenic regulatory factors.

CRISPR-Cas9 was used to engineer *Melanocortin receptor-1 (MC1R)* knock-out mutants in Oujiang color common carp (Mandal et al., 2020). These mutants exhibited altered melanophore production, resulting in grayish or albino skin patches. This result suggests that *MC1R* plays a vital role in melanogenesis in fish. In another study, two Agouti signaling protein (ASIP) genes were targeted by CRISPR/Cas9 technology in Oujiang color common carp and found that they are involved in regulating melanin distribution and aggregation during the formation of black patches on the skin (Chen et al., 2019). Similarly, CRISPR-Cas9 was used to target the *Tyrosinase (TYR)* gene in white crucian carp (WCC) and its hybrid progeny (WR) (Liu et al., 2019). They successfully reduced TYR protein levels, leading to a reduction in melanin and altered pigmentation in the fish. Additionally, key pigment synthesis genes were downregulated in the mutant fish, highlighting the role of tyr in melanin synthesis.

In gibel carp, CRISPR/Cas9 disrupted *foxl2* gene variants and demonstrated differences in their expression, leading to significant effects on ovary development and germ cell depletion (Gan et al., 2021). This work sheds light on gene diversification and evolution in polyploid gibel carp. Recently, CRISPR/Cas9 gene editing targeted two runt-related transcription factor 2b (runx2b homeologs), that play a key role in the development of intermuscular bones (IBs) in gibel carp (Gan et al., 2023). The study demonstrates that simultaneous disruption of both genes led to the complete loss of IBs. In a recent breakthrough, scientists successfully employed genome editing techniques to modify the *bmp6* gene in diploid crucian carp (Kuang et al., 2023). This achievement marks a significant milestone as it led to the creation of a specialized strain of crucian carp devoid of intermuscular bones (IMBs). The edited strain exhibited accelerated growth rates when compared to their wild-type counterparts, opening new possibilities for genome editing applications in other cyprinid fish species to eliminate IMBs.

This technique holds promise for creating models to study immune response, and it represents the first evidence of CRISPR/Cas9’s high efficiency in targeted gene-disruption through homologous recombination (HR). The study in rohu carp demonstrates the successful application of the CRISPR/Cas9 system to disrupt the *Toll-like receptor 22 (TLR22)* gene, marking a notable advance in teleost fish genetics (Chakrapani et al., 2016). In grass carp, CRISPR/Cas9 genome editing was used to target and knockout the *gcJAM*-A (Junctional Adhesion Molecule-A) gene (Ma et al., 2018). This work reduced grass carp reovirus (GCRV) infection in permissive grass carp kidney cells (CIK), showing promise for controlling the disease in aquaculture.

Therefore, recent studies demonstrate the successful use of genome editing, particularly CRISPR/Cas9, in cyprinid species for trait improvement. The availability of reference genomes, which will allow better design of guide RNAs for the CRISPR-Cas9 vector, resulting in minimized off-target effects. These techniques hold great potential for targeting complex traits like immunity and disease resistance, crucial for aquaculture improvements and disease management.

## 8 Future perspectives and concluding remarks

**FIGURE 3:**
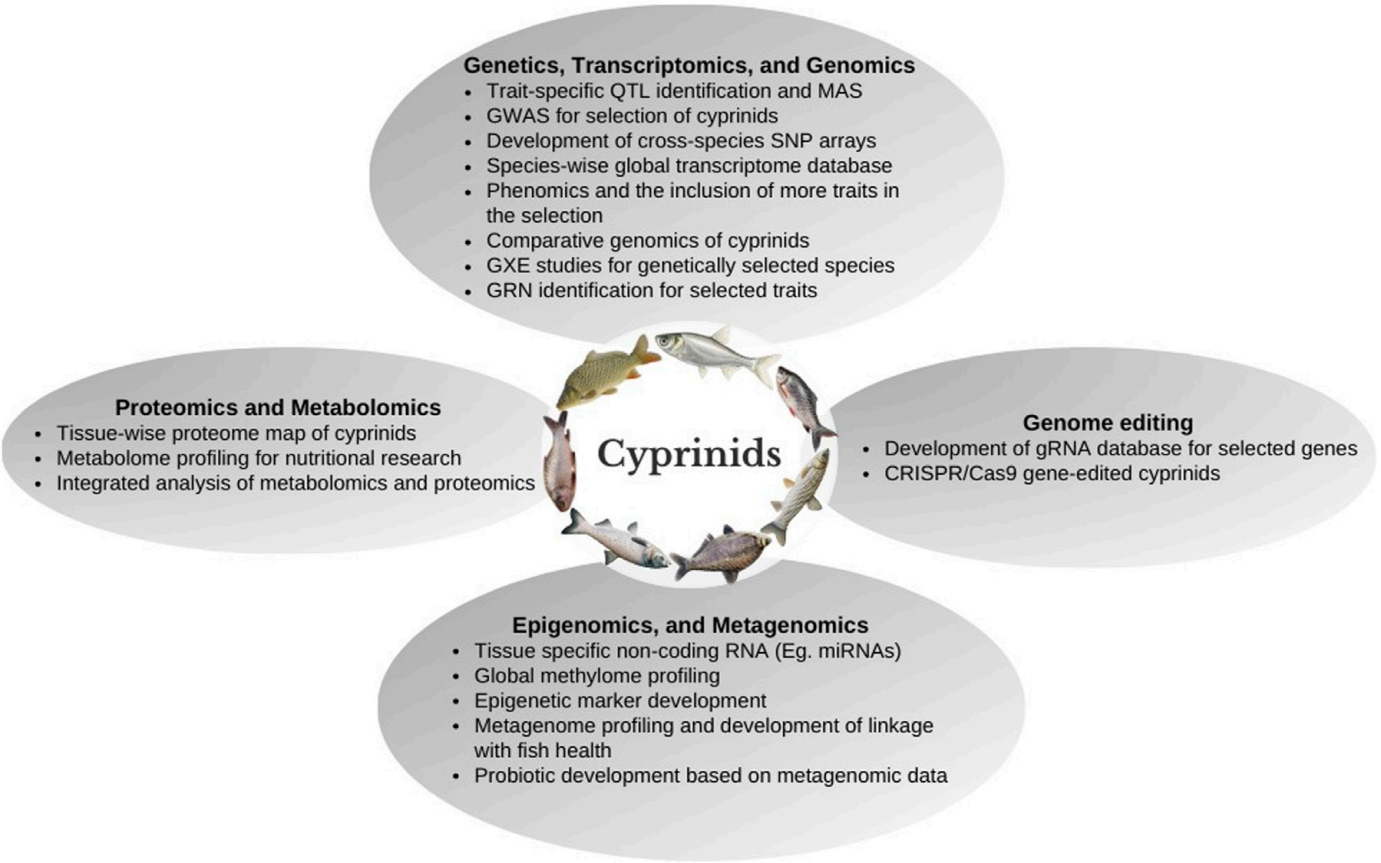
Future perspectives of cyprinids in aquaculture.

Achieving sustainable aquaculture production depends on utilizing a diverse range of genetic and genomic resources. We have comprehensively highlighted future perspectives and way forward in cyprinid species for increasing aquaculture production in Figure 3. The selective breeding program in cyprinid species has successfully improved traits like growth rate and disease resistance. Nevertheless, striking a balance between maintaining high selection intensity and keeping low inbreeding rates remains a long-term challenge. Some breeding programs failed due to a weak base population or limited stocks involved, leading to inbreeding and loss of genetic viability. For successful selective breeding in cyprinid species, it is essential to generate a comprehensive set of heritability values and genetic correlations for important traits. Overlooking genotype-by-environment interactions may result in a range of phenotypes, leading to a shortfall in the desired performance of genetically selected cyprinid species. Therefore, selection strategies should consider GxE interactions during cyprinid breeding programs. Both the Intergovernmental Panel on Climate Change (IPCC) and the Food and Agriculture Organization (FAO) have issued warnings about the severe impact of climate change on global warming and its implications for the aquaculture sector (Cochrane et al., 2009). As a result, it is essential to identify adaptive genes or traits in cyprinid species that can thrive in changing environmental conditions and disease outbreaks.

In cyprinid species aquaculture, the major concern is feed cost, which constitutes 50%–60% of the total production cost. Therefore, enhancing feed use efficiency through selective breeding is essential to reduce input costs in aquaculture. Accurate estimation of FCR traits and feed use efficiency is challenging in fish due to their rearing environment, and incorrect methodologies in many studies, resulting in low heritability estimates. As a solution, an indirect method of selection, such as measuring growth during feeding/re-feeding, could be used to improve these traits in cyprinids. In addition to feed-use efficiency, the selective breeding program for cyprinid species should also consider other important traits like tolerance to temperature, stress, or salinity to achieve genetic improvement. However, implementing and disseminating genetic improvement programs in developing countries face serious constraints, including limited financial resources, infrastructure, and skilled manpower.

The integration of genomic resources into cyprinid species genetic improvement programs is in the early stages. Whole genome sequences and phenotypic data are important for genomic selection. It is crucial to validate markers linked to production and performance traits using larger sample sizes and efficient genotyping methods. Cyprinid species genome sequences are often in draft form, requiring the generation of annotated databases with functional assignment. Combining sequenced reads to create a reference genome for each cyprinid species is essential. Also, various gene transfer techniques were used for genetic improvement and manipulation in aquatic species. Looking at the ecological concerns, sterile transgenic fish need to be produced through ploidy manipulation or hormonal treatment for trait improvement in carps. Investigating the function of predicted genes through expression studies or gene knock-out approaches, like CRISPR/cas9, can further enhance genetic improvement in aquaculture industries. Molecular markers play a crucial role in some fish species’ selective breeding programs, enabling the construction of linkage maps linked to economically important traits. It is imperative to identify genome-wide high-density markers associated with key traits, including disease resistance, feed conversion efficiency, reproductive fitness, and responses to abiotic stressors like temperature, dissolved oxygen, and salinity. Although medium or high-density SNP arrays have been reported for some carp species, the adoption of techniques like restriction-site associated DNA sequencing (RAD-Seq) and genotyping by sequencing (GBS) is essential to generate SNP arrays in other cyprinid species that are relevant to the traits of interest. These advanced genotyping methods will aid in the identification and utilization of valuable genetic variations in cyprinid breeding programs. It is estimated that an SNPs array can increase an average of 25% genetic gain with high selection accuracy in aquaculture species (Allal and Nguyen, 2022).

Polymorphic markers enable high-density linkage maps, while QTL for production traits needs to be identified and validated in cyprinid species. Genomic selection requires genotypic and phenotypic data for accurate GEBV prediction. Reliable phenotypic data is crucial for estimating genetic parameters and improving selection accuracy and genetic gain. GxE relationship data aids in precise genomic selection in cyprinid species. Understanding epigenetic regulation in cyprinid species is limited but important for estimating genetic variation and gene expression modulation. Climate change and environmental factors affect traits involving epigenetic regulation through DNA methylation or miRNAs. Further research on DNA methylation’s mechanism and association with an important traits in cyprinid species is necessary. Epigenome editing can establish causal relationships between epigenetic factors and traits of interest.

To understand complex biological processes and traits in cyprinids, integrating multi-omics data (transcriptome, epigenome, proteome, metabolome, etc.) is crucial. Understanding the gene-regulatory network for growth and disease resistance aids cyprinid aquaculture. Additionally, each cyprinid species should have an integrated database for assessing genomic, proteomic, expression profiles, SNP/microsatellite markers, and linkage mapping data. The tissue-specific proteome data needs to be generated to decipher the key role of expressed proteins in particular situations. This database facilitates comparative genomics and key gene mapping for production traits and aids in population dynamics and fisheries management. The cost of sequencing and GBS has significantly reduced, enabling broader application. However, a downstream analysis may face challenges in dealing with genome complexity as the cost continues to decrease.

Genomic selection has been suggested to yield increased genetic gain with high selection accuracy. However, its implementation in cyprinid species breeding programs is still in its early stages and requires further exploration. Many academic and research institutes have developed genomic resources for important cyprinid species, but greater collaboration between academia and industry is needed to fully harness their potential for the genetic improvement of regional cyprinid species. Looking ahead, growing carp genetics and genomics data can enhance selection accuracy, production, and sustainability in aquaculture. Genomic selection will be pivotal in future cyprinid species breeding programs, surpassing traditional pedigree-based selection methods in terms of accuracy.

In conclusion, this review provides comprehensive information on genetic and genomic progress achieved in cyprinid species. Although selective breeding techniques achieved considerable progress in carp genetic research, further genomic selection holds a promising approach for enhancing the accuracy of breeding values. NGS, TGS (Third-Generation Sequencing) and computational tools have revolutionized the understanding of carps and other species biological research and generated a vast amount of genomic resources. This has further led to molecular-level studies concerning disease resistance, growth, reproduction, and adaptation to changing environments in cyprinids. This review depicts the progress of genomics in important cyprinid species at the level of genome, transcriptome, proteome, metabolome, metagenome, epigenome, etc. Gene transfer and genome editing applications steadily expanding across various aquaculture species, including carps, for genetic improvement and understanding of biological functions. This review serves as a vital resource for carp aquaculture research communities.
