# Supplementary material for: Genetic improvement and genomic resources of important cyprinid species: status and future perspectives for sustainable production
Source: Front Genet. 2024 Sep 19;15:1398084. doi: 10.3389/fgene.2024.1398084 (PMC11446788; doi:10.3389/fgene.2024.1398084)
Supplement: Supplementary file 1 [file Table1.DOCX]

**Supplementary Table S1. DNA methylation and microRNAs studies in cyprinid species.**

| **Sr. No** | **Species** | **Tissue** | **NGS Platform Used** | **No of DMR and DMGs identified /miRNAs identified** | **Purpose of study** | **Reference** |
| --- | --- | --- | --- | --- | --- | --- |
| 1. | Common Carp (*Cyprinus carpio*) | Milt | Illumina | 1305 and 1729 DMRs | Understand the aging of sperm on the fertilization performance | (Cheng et al., 2021) |
|  |  | Blood | Illumina Genome Analyzer | 394 potential miRNA binding sites in 206 target mRNAs were predicted for 83 miRNAs | Analysis of the miRNA-related SNPs and their effects provided insights into the effects of SNPs on miRNA biogenesis and function | (Zhu et al., 2012) |
|  |  | Juvenile gonad | Illumina/  Solexa Genome Analyzer | 4,443 miRNAs (3795 existing miRNAs, and 648 conserved miRNAs) | Atrazine can up-regulate aromatase expression through miRNAs, which supports the hypothesis that atrazine has endocrine-disrupting activity | (Wang et al., 2019a) |
|  |  | Skeletal muscle | Solexa | 188 known conserved miRNAs; 7 novel miRNAs | First identification and profiling of miRNAs related to the muscle biology of the common carp | (Yan et al., 2012) |
|  |  | Liver | Illumina Hiseq2500 | 698 miRNAs | miRNA expression profile of the liver of common carp infected with *Flavobacterium columnare* will pave the  way for the development of effective strategies to fight against FC infection | (Zhao et al., 2016) |
|  |  | Skin | Solexa | 13 miRNAs differentially expressed in red and white skin | Provide a novel insight into the determination of skin color in fish. | (Yan et al., 2013) |
| 2. | Crucian carp (*Carassius carassius* *L.*) | Skin | Illumina HiSeq 2500 | 7961 DMRs | Molecular mechanisms underlying skin color variations | (Zhang et al., 2017) |
| 3. | Common carp (*Cyprinus carpio* *L.*). | Brain and gonad | Single-cell bisulfite sequencing (scBS-seq) | - | Revealed potential application of extenders in storage of sperm in common carp breeding programs | (Cheng et al., 2023) |
| 4. | *Carassius auratus red var.* and *Cyprinus carpio L* allotropids | Erythrocytes | NovaSeq 6000 | 905 DMGs; 258 miRNA | It helps in identifying the regulatory network between DNA methylation and miRNAs in allotriploids. | (Ren et al., 2022a) |
| 5. | Grass carp (*Ctenopharyngodon idellus*) | Spleen | Illumina HiSeq X Ten | 6214 DMRs and 4052 DMGs | Revealing age-dependent grass carp reovirus (GCRV) susceptibility | (He et al., 2022b) |
|  |  | Liver | Illumina Hiseq 2000 | 2048 DMGs | Helps in understanding of the key genes and pathways that affect the immune system at different development stages | (Gui et al., 2022) |
|  |  | Blood | Illumina Hiseq 4000 | 20010 DMGs in promoter; 27016 DMGs in gene body | Identified four selection signature that include growth and metabolism, immunity, foraging and learning behaviours | (Li et al., 2021b) |
|  |  | Spleen | Illumina HiSeq 4000 | 324 known conserved miRNAs; 9 novel miRNAs | Determined the miRNA transcriptome as well as miRNA-mRNA interaction networks in normal spleen tissue during the late development stages of grass carp. | (Zhao et al., 2022) |
|  |  | Kidney | Solexa | 61 known conserved miRNAs; 116 novel miRNAs | 1. Characterize the expression of miRNA in the grass carp in relation to MAS;  2. Evaluate the diagnostic potential of the investigated miRNAs as biomarker for MAS. | (Xu et al., 2016) |
|  |  | Brain, muscle, gill,  intestine, and heart | Illumina HiSeq X Ten | 1,506 miRNA precursors | Provide novel insights into the genesis and development of  microRNA clusters in teleost. | (Niu et al., 2023) |
|  |  | Kidney | Illumina HiSeq 2000 | 188 miRNAs | Demonstrates the feasibility of identifying miRNA targets by transcriptome analysis. | (Xu et al., 2014c) |
| 6. | Silver Carp (*Hypophthalmichthys molitrix*) | Heart, brain, liver, gill | Illumina Noveseq | 229 known miRNAs; 391 putative novel miRNAs | This study useful for understanding the regulatory mechanism of miRNAs in silver carp under hypoxia stress | (Wang et al., 2021b) |
| 7. | Bighead Carp (*Hypophthalmichthys nobilis*) | Larvae | Illumina Hiseq X Ten | 1046 miRNAs | Revealed the repertoire of miRNAs that are active during early development of bighead carp. | (Fu et al., 2022) |
| 8. | Silver Carp (*Hypophthalmichthys molitrix*), Bighead Carp (*Hypophthalmichthys nobilis*) | Heart, liver, brain, spleen and  Kidney | Illumina Genome  Analyzer (GPL9330) | 167 (Bighead carp) and 166 (Silver carp) conserved miRNAs | Duplication of animal miRNA genes may occur through evolutionary processes which are similar to the protein-coding genes. | (Chi et al., 2011) |
| 9. | Rohu (*Labeo rohita*) | Liver | IlluminaNextSeq 500 | 138 conserved miRNAs; 161 novel miRNAs | First report of the presence of miRNAs in liver tissue of rohu and their comparative profile linked with metabolism serves as a vital resource as a biomarker | (Rasal et al., 2020c) |

**Supplementary Table S2. Metagenomic studies in cyprinid species.**

| **Sr. No** | **Species** | **NGS Platform Used** | **Major Phyla identified** | **Environmental variable/challenge/purpose** | **Purpose of study** | **Reference** |
| --- | --- | --- | --- | --- | --- | --- |
| 1. | Common carp (*Cyprinus carpio*) | Proprietary sequencing platform of BGI Genetics | Proteobacteria, Bacteroidetes, Fusobacteria,  Firmicutes, and Actinobacteria | Toxicity (Norfloxicin) | Environmental concentrations of NOR (Norfloxacin) in common carp that alter the composition, structure, and abundance of ARGs (Antibiotic resistance genes) in the gut microbiota | (Cheng et al., 2023) |
|  |  | Illumina NextSeq 550 | Proteobacteria, Actinobacteria,  Bacteroidota, Firmicutes, Cyanobacteria, Planctomycetota | Skin mucus microbiome | It showed a high level of host genome contamination | (Papp et al., 2023) |
|  |  | NovaSeq 500 | Arenaviridae (49%), Hepeviridae (20%), Chuviridae (21%), Astroviridae (3%), Flaviviridae (2%) | Invasion of carp in Australian waters | It marked the lack of viruses between invasive and native fish that revealed little tramission of virus to native species from common carp | (Costa et al., 2021) |
|  |  | NovaSeq 6000 | Enterobacteriaceae, Pseudomonadaceae, Aeromonadaceae, Streptococcaceae, Campylobacteraceae, Enterococcaceae | Antibiotic Resistant Genes | Reveals possible relationship between the occurrence of acquired ARGs in domestic and wild animal populations | (Libisch et al., 2022) |
|  |  | Novogene | Fusobacteria (80%),  Bacteriodetes (15%), Firmicutes (3%) | Community structure of Common carp | Identified around 19 types of bacterial phyla | (Nugrahi et al., 2021) |
| 2. | Crussian carp (*Carassius carassius*) | Illumina MiSeq | Cetobacterium, Aeromonas, and Plesiomonas | Environmental adaptation | Significant differences in the relative abundances of different bacterial phyla in the different environments | (Zhang et al., 2019b) |
| 3. | Grass carp (*Ctenopharyngdon idella*) and crucian carp (*Carassius carassius*) | Illumina HiSeq 2500 | Bacteroidetes, Actinobacteria, Cetobacterium_sp. | Comparative analysis in new lineages | differences in gut bacterial community composition may be an important factor contributing to the rapid growth and high disease resistance of the new fish lineages. | (Zou et al., 2020) |
| 4. | Common carp (*Cyprinus carpio*), Silver carp ( *Hypophthalmichthys molitrix*), Bighead carp ( *Hypophthalmichthys nobilis*) | Illumina HiSeq 2000 | Proteobacteria, Firmicutes,  Fusobacteria | Compared the fecal microbiomes | Comparison of wild- and lab-invasive carps revealed five shared OTUs (Operational taxonomic unit) that comprised approximately 40 % of the core fecal microbiome. | (Eichmiller et al., 2016) |
| 5. | Gold fish (*Carassius auratus*) | Illumina HiSeq 4000 | Gemmobacter, Bosea, Rhizobium, Shinella | Microplastics pollution | Understanding of the interaction mechanism between APs ( Antimicrobial pharmaceuticals) and antibiotics in real aquatic environment | (Zhang et al., 2022a) |
| 6. | Rohu (*Labeo rohita*), Catla  (*Labeo catla*) and mrigal (*Cirrhinus mrigala*) | IonTorrent Personal Genome Machine | Proteobacteria (15–40%), Firmicutes (16–21%), Actinobacteria (18–34%), Bacteroidetes (6–19%) | Polyculture system | Gut microbiome might have resulted from niche partitioning and selective pressures that could be species-specific | (Mukherjee et al., 2020a) |
| 7. | Grass carp (*Ctenopharyngdon idella*) | Illumina MiSeq | Aeromonas, Vibrio, Pseudomonas | Yeast culture dietary supplementation | Beneficial effects of YC (Yeast culture) feeding on gut microbiota, growth and biochemical parameters | (Liu et al., 2018) |
|  |  | Illumina HiSeq 2000 | Firmicutes, Proteobacteria, Fusobacteria | Factors affecting the gut microbiome | Metabolic role played by the gut microbiome in grass carp can be affected by feeding | (Ni et al., 2014a) |
| 8. | Silver carp (*Hypophthalmichthys molitrix*); Bighead carp (*Hypophthalmichthys nobilis*); Grass carp (*Ctenopharyngodon idella*); Common carp (*Cyprinus carpio*) | Illumia Miseq | Proteobacteria, Firmicutes, Bacteroidetes, Fusobacteria | Comaparitive study | Gut microbiotas of Asian carp depend on the exact species, even when the different species were cohabiting in the same environment | (Li et al., 2018) |

**Supplementary Table S3. Molecular markers identified in carps.**

| **Species** | **Marker Type** | **No. of markers** | **Stock/Sample collection** | **Sample size** | **Trait targeted / Purpose** | **Reference** |
| --- | --- | --- | --- | --- | --- | --- |
| Black carp (*Mylopharyngodon piceus*) | Microsatellite | 31 | Yangtze River basin | 269 | To provide novel molecular tools for studies of population genetic diversity and new evidence of genetic differentiation between wild and cultured populations of black carp | (Zhou et al., 2020) |
| Bighead carp (*Hypophthalmichthys nobilis*) & Silver carp (*H.molitrix*) | Microsatellite | 924 | Yangtze River, China | 198 | To construct a genetic linkage map based on SNP (2b-RAD) and microsatellite markers in bighead carp  To facilitate QTL mapping for sex in bighead carp and silver carp | (Zhou and Gui, 2018) |
|  | SNP | 2976 |  |  |  |  |
|  | SNP | SNP (57 nuclear & 1 mitochondrial) | Mississippi River Basin (MRB) | 2798 | To genetically identify putative hybrids and to characterize the extent of interspecific gene flow | (Lamer et al., 2015) |
| Common Carp (*Cyprinus carpio*) | Mitochondrial (mt) marker | Mitochondrial 12S ribosomal RNA (12S rRNA), tRNA-Val, 16S ribosomal RNA (16S rRNA), cytochrome b (cyt b), & control region (CR) sequences | Centre of Carpathian Basin (Hungary) | n= 38 from 13 strains | To assess the genetic diversity, population structure & phylogenetic relationship | (Toth et al., 2022) |
|  | Microsatellite marker | 12 | Carpathian Basin, Hungary | 630 | Evaluate genetic diversity and structure of 13 Hungarian common carp strains & Amur wild carp | (Tóth et al., 2020b) |
|  | Microsatellite | 250 | Songpu Aquaculture Experimental Station, Heilongjiang River Fisheries Research Institute | 46 - 190 | To locate QTLs on linkage groups  To identify common & overlapping QTLs associated with 3 growth-related traits  To investigate genetic architecture underlying growth-related traits | (Lv et al., 2016) |
|  | SNP | 7839 | Yellow River carp population | 82 | To conduct a Genome-wide association study (GWAS) and association mapping using the common carp 250 K SNP genotyping array to analyze genome-wide SNPs. | (Zhou et al., 2018) |
| Grass Carp (*Ctenopharyngodon idella*) | SNP | 368 | China | 500 | To identify changes in gene and genotype frequencies at four early developmental stages including hatching, first feeding, juvenile fish, and young fish) | (He et al., 2018) |
|  | SNP | 7 in CD40 &  5 in CD154 | Duofu fish farm (Wuhan, China) | Resistant (n=70)  Susceptible  (n= 64) | To identify SNPs associated with resistance of grass carp to GCRV (Grass carp reovirus) | (Lu et al., 2018) |
| Indian Major Carps (IMC)  *Labeo catla*  *Labeo rohita,*  *Cirrhinus mrigala* | Mitochondrial (mt) marker | cytochrome b  ATPase 6/8 | River Mahanadi | 90 | To understand the evolutionary pattern of mt DNA genes in carps  To explore the phylogeographic structure of IMC | (Das et al., 2013a) |
| *Rohu (Labeo catla)* | Mitochondrial (mt) marker | Cytochrome C Oxidase 1 | River Chenab, (Punjab & Pakistan) | 15 | Molecular-based identification of Catla using CO1  Estimation of genetic distances & evolutionary tree | (Naeem et al., 2020) |
|  | SNP | 3048 | Halda, Jamuna and Padma rivers in Bangladesh | 900 | To assign putative sibship to, ‘candidate founders’ of breeding population  To investigate the level of genetic relatedness and molecular genetic diversity within and among catla samples. | (Hamilton et al., 2019a) |
|  | silicoDArT markers | 4726 |  |  |  |  |
|  | Mitochondrial (mt) marker | 2 | Illinois River,  Wabash River,  Missouri River, Mississippi River | 309 | To apply population genetics, phylogenetic analysis, & biological knowledge of genetic variation in Silver carp & its cyprinid relatives to understand invasion dynamics | (Stepien et al., 2019) |
|  | Microsatellite | 10 |  |  |  |  |
| Silver carp (*Hypophthalmichthys molitrix*) | SNP | 3,134 | Yangtze River, China | 198 F1 individuals sequencing | To detect QTL assosicated with growth-related traits (body length, body height, head length and body weight) at 6, 12 and 18 months post hatch (mph) | (Wang et al., 2019b) |
